# Electroacupuncture Prevents TBI-Induced Synaptic Loss by Inhibiting CaMKII/Drp1-Dependent Mitochondrial Fission

**DOI:** 10.3390/biom16081169

**Published:** 2026-08-11

**Authors:** Sisi Zhao, Luxi Cao, Feidan Deng, Zhenge Liao, Xiaoxiang Li, Guanglei Li, Chunzhi Tang, Yimin Zhang, Shujun Lin

**Affiliations:** 1 Medical College of Acupuncture-Moxibustion and Rehabilitation, Guangzhou University of Chinese Medicine, Guangzhou 510006, China; zhaosisi804@163.com (S.Z.); 15973861661@163.com (Z.L.); andy4sean@163.com (X.L.); jordan664@163.com (C.T.); 2 School of Traditional Chinese Medicine, Jinan University, Guangzhou 510632, China; caoluxi@jnu.edu.cn (L.C.); dengfeidan@stu.jnu.edu.cn (F.D.); guanglei0719@163.com (G.L.)

**Keywords:** electroacupuncture, traumatic brain injury, synaptic loss, mitochondrial fission, CaMKII/Drp1

## Abstract

(1) Background: Traumatic brain injury (TBI) triggers synaptic loss, leading to long-term neurological deficits. Electroacupuncture (EA) benefits neurological conditions, but its mechanisms after TBI remain unclear. (2) Methods: We used a controlled cortical impact (CCI) mouse model. Behavioral outcomes were assessed using the modified neurological severity score (mNSS), rotarod, Y-maze, and novel object recognition test (NORT). Synaptic morphology was examined by transmission electron microscopy (TEM). Energy metabolism was assessed using biochemical assays, and mitochondrial function was assessed using flow cytometry. Quantitative real-time PCR (qPCR) and Western blotting (WB) were used to examine the underlying molecular mechanisms. (3) Results: We found that EA ameliorates TBI-induced motor and cognitive impairments by preserving synaptic and mitochondrial integrity. EA-treated mice showed improvements in mNSS, rotarod, NORT, and Y-maze performance, along with preserved synaptic ultrastructure and mitochondrial function. CaMKII overexpression abolished EA-induced neuroprotection, identifying the CaMKII/Drp1 axis as a key mediator. (4) Conclusions: Thus, EA limits TBI deficits by restraining CaMKII/Drp1-driven mitochondrial fission and subsequent synaptic loss.

## 1. Introduction

Traumatic brain injury (TBI) remains one of the most prominent contributors to global mortality and long-term neurological disability, with survivors often facing persistent motor and cognitive sequelae that severely undermine daily functioning and independence [1,2,3]. Neuropathological evidence consistently implicates the hippocampus and cerebral cortex as primary sites of damage, a pattern that correlates strongly with the observed behavioral deficits in both clinical and experimental settings [4]. Despite decades of research, conventional pharmacological and surgical interventions have failed to produce consistent functional recovery, leaving a considerable therapeutic void [5]. In response, preclinical efforts have increasingly turned to neuromodulatory strategies, among which electroacupuncture (EA) has emerged as a particularly active area of investigation [5,6,7]. Unlike single-target agents, EA concurrently modulates inflammatory cascades, oxidative stress, and apoptotic signaling—mechanisms that are mutually reinforcing in the post-TBI milieu [5,8]. This broad mechanistic coverage, while not fully elucidated, offers a rational basis for its application in a pathology as inherently multifaceted as TBI [5,8].

Numerous studies have established that synapse loss is among the earliest events in the neurodegenerative cascade following TBI, and its extent serves as a robust predictor of long-term functional outcomes [5,6,7]. TBI induces significant synaptic loss not only in the perilesional cortex but also in distal brain regions, particularly the hippocampus. For instance, a 60% loss of synapses in the hippocampal CA1 region has been reported as early as 2 days post-injury, with progressive region-specific loss occurring in the hippocampus and other areas distal to the injury site [6,7,9]. Of note, synaptic loss can also occur in the contralateral hemisphere at chronic stages [10,11,12]. These patterns vary across brain regions and time points, suggesting region-dependent vulnerability after TBI [11]. At the circuit level, the erosion of synaptic contacts disrupts information flow across cortical and hippocampal networks, a disturbance that not only facilitates subsequent neuronal death but also directly underpins the characteristic motor planning deficits and memory retrieval failures observed in injured subjects [7,9,13]. Importantly, this synaptic degeneration appears to represent a convergent endpoint for multiple interdependent injury mechanisms: reactive oxygen species overproduction, impaired ATP synthesis, and sustained microglial activation have all been shown to converge on common synaptic substrates, ultimately driving the transition from molecular derangement to clinically measurable disability [12,14,15,16]. In controlled cortical impact (CCI) models, hippocampal synaptophysin labeling is reduced by approximately 30% as early as 72 h post-injury, with similar decrements observed in both ipsilateral and contralateral hippocampus, and this synaptic loss represents a critical secondary injury mechanism [7,17].

Mitochondrial dynamics are critical for sustaining synaptic integrity, and their disruption has emerged as a central pathogenic mechanism in TBI [18,19]. Under physiological conditions, neurons maintain a tightly regulated equilibrium between mitochondrial fusion and fission to match regional ATP demands and support vesicular trafficking at presynaptic terminals [19]. This balance is severely disrupted following mechanical insult, primarily through aberrant activation of dynamin-related protein 1 (Drp1), which shifts the equilibrium toward excessive fission and results in widespread mitochondrial fragmentation [19,20]. The accumulation of fragmented organelles can impair mitophagic clearance and respiratory chain efficiency, culminating in ATP depletion and ionic dysregulation—a deteriorating cascade that has been implicated not only in TBI but also across a spectrum of neurodegenerative disorders [18,19].

Parallel to this fission-driven pathology, TBI triggers a sustained elevation of intracellular Ca^2+^, which in turn activates Ca^2+^/calmodulin-dependent protein kinase II (CaMKII), a multifunctional kinase with well-established roles in long-term potentiation and learning [21]. Once phosphorylated, CaMKII directly phosphorylates Drp1 at the Ser616 residue, promoting its mitochondrial translocation and potentiating fission activity [22,23]. This CaMKII–Drp1 signaling axis has been established as a critical driver of mitochondrial fragmentation across multiple pathological contexts, including ischemia–reperfusion injury and excitotoxic damage, where it directly links calcium overload to mitochondrial fission and downstream cellular dysfunction [24]. Although direct evidence in TBI models remains limited, the mechanistic conservation of this pathway across different injury paradigms supports its likely role in TBI-induced mitochondrial pathology, offering a plausible molecular link between acute excitotoxic stress and the ensuing synaptic loss, bioenergetic failure, and oxidative burden.

Previous studies have demonstrated that acupuncture interventions in TBI models can modulate diverse biochemical cascades, including histone deacetylase overexpression and aberrant BDNF-related Akt/GSK-3β signaling [25], regulate the TLR2/4–NF-κB inflammatory axis [26], suppress the PANX1/ATP/Ca^2+^ pathway [8], and restore autophagic flux [27]. Despite this broad mechanistic portfolio, the specific actions of EA on mitochondrial fission events and their contribution to synaptic loss have not been systematically addressed. To fill this gap, the current study was undertaken to test the hypothesis that EA confers neuroprotection in the pericontusional cortex—at least in part—by counteracting CaMKII/Drp1-driven mitochondrial fragmentation and mitigating accompanying ultrastructural damage to synaptic terminals.

Given the mechanistic centrality of CaMKII/Drp1-driven mitochondrial dysregulation in TBI pathophysiology, we considered EA a promising candidate for therapeutic exploration. Accumulating clinical and preclinical evidence supports the efficacy of EA in various central nervous system disorders, including ischemic stroke, Alzheimer’s disease, and major depressive disorder [28,29]. More specifically, in TBI settings, EA has been repeatedly associated with reduced coma duration, lowered mortality rates, and accelerated cognitive recovery [30]. Our own previous investigations have further shown that EA alleviates post-TBI neurofunctional deficits, an effect we attributed, at least in part, to the modulation of neuronal autophagy and glial-mediated inflammatory responses [26,29]. Despite these observations, the downstream molecular events through which EA preserves synaptic architecture remain to be fully clarified. Given the well-documented interconnection between mitochondrial fission/fusion balance and synaptic viability, we postulated that EA may exert its protective effects, at least partially, via interference with the CaMKII/Drp1 phosphorylation cascade. Accordingly, the present study was designed to test whether EA improves both motor and cognitive outcomes in TBI mice by attenuating excessive CaMKII/Drp1-mediated mitochondrial fragmentation and mitigating the consequent structural erosion of synapses.

## 2. Materials and Methods

### 2.1. Animals

Male C57BL/6J mice (6–8 weeks old, weighing 22–25 g) were obtained from Guangdong Zhiyuan Biopharmaceutical Technology Co., Ltd. (Guangzhou, China). Animals were housed in standard polypropylene cages under controlled environmental conditions (temperature 24 ± 1 °C, relative humidity 50 ± 5%, 12 h/12 h light–dark cycle, lights on at 07:00), with rodent chow and tap water available ad libitum. All experimental procedures were approved by the Animal Ethics Committee of Guangzhou University of Chinese Medicine (Approval No. 20250708011, dated 8 July 2025) and were conducted in accordance with the National Institutes of Health Guide for the Care and Use of Laboratory Animals.

### 2.2. CCI Model Establishment

TBI was induced using a controlled cortical impact (CCI) device (Model YHCI99, Wuhan Yihong Technology Co., Ltd., Wuhan, China), following established protocols. Briefly, mice were anesthetized with isoflurane and secured in a stereotaxic frame. A 4 mm circular craniotomy was performed at coordinates 1 mm posterior to bregma and 1 mm lateral to the midline, with the dura mater left intact. Cortical contusion was then delivered using an impactor equipped with a 3 mm diameter tip, set to the following parameters: impact velocity of 3.5 m/s, compression depth of 1.5 mm, and dwell time of 250 ms [31]. Immediately after impact, bleeding was controlled, the scalp incision was sutured, and the wound was disinfected. Mice were placed on a thermostatically controlled heating pad to maintain core body temperature at 37 ± 0.5 °C throughout the recovery period until full emergence from anesthesia.

Neurological function was assessed using the modified neurological severity score (mNSS) at baseline and on post-injury day 3, and only animals with scores ranging from 7 to 12 were assigned to subsequent experiments. The overall modeling success rate exceeded 90%, with fewer than 10% of animals excluded—primarily due to low body weight compounded by poor anesthetic tolerance, or the presence of intracranial hematoma. No epileptiform events were observed during the entire experimental course. Importantly, all exclusions were made before group assignment, based on predefined objective criteria, and the remaining animals were randomly allocated to treatment groups.

### 2.3. Electroacupuncture Treatment

EA treatment commenced at 3:00 p.m. on the day of TBI induction (approximately 2 h post-injury) and was repeated once daily at the same time (24 h inter-session interval) for three consecutive days. During each session, sterile acupuncture needles (0.16 mm diameter × 7 mm length) were manually inserted into the Baihui (GV20) and Fengfu (GV16) acupoints [26,29], and then connected to an electroacupuncture device (HANS-200A, Nanjing Jisheng Medical Technology Co., Ltd., Nanjing, China). Stimulation parameters were set to a constant current of 0.1 mA at a frequency of 2 Hz, with each session lasting 15 min. All EA procedures were performed by the same experienced operator to ensure consistency across sessions.

### 2.4. Animal Experiments

This study comprised three independent animal cohorts, each designed to address a distinct research objective.

Experiment 1 was conducted to evaluate the therapeutic efficacy of EA on motor and cognitive functions in TBI mice, and to explore its mechanistic correlates. Animals were assigned to three groups (*n* = 20 per group): (1) Sham group (craniotomy without cortical impact or EA); (2) TBI group (injury alone); and (3) EA group (TBI followed by EA intervention).

Experiment 2 incorporated a viral vector control to rule out non-specific effects arising from the stereotaxic injection procedure itself. This cohort comprised six groups (*n* = 14 per group): (1) TBI; (2) TBI + AAV-scrambled shRNA (scrambled sequence control for AAV-shCaMKII); (3) TBI + AAV-shCaMKII (CaMKII knockdown); (4) EA; (5) EA + AAV-mCherry (empty vector control for AAV-CA-CaMKII); and (6) EA + AAV-CA-CaMKII (CaMKII overexpression under EA treatment).

Experiment 3 was specifically designed to interrogate the causal contribution of CaMKII-mediated mitochondrial fission to the neurofunctional recovery conferred by EA after TBI. This cohort included four groups (*n* = 25 per group): (1) TBI; (2) TBI + AAV-shCaMKII (CaMKII knockdown); (3) EA; and (4) EA + AAV-CA-CaMKII (CaMKII overexpression under EA treatment).

For both overexpression and knockdown paradigms, all AAV vectors were prepared at identical titers and injected at equal volumes to ensure comparable vector loads across groups.

### 2.5. Intra-Cerebroventricular (ICV) Injection

All adeno-associated viral (AAV) constructs used in this study were obtained from BrainVTA Co., Ltd. (Wuhan, China). Viral injections were performed four weeks prior to TBI induction. Briefly, mice were anesthetized with isoflurane and positioned in a stereotaxic frame. A 2 μL aliquot of AAV suspension was delivered intracerebroventricularly into the lateral ventricle ipsilateral to the injury site using a Hamilton microsyringe, at the following coordinates relative to bregma: anteroposterior (AP) −0.3 mm, mediolateral (ML) +1.0 mm, and dorsoventral (DV) −3.0 mm, according to the mouse brain atlas [32]. The injection was performed at a constant rate over 3–5 min, after which the needle was left in place for an additional 10 min before slow retraction to minimize reflux of the viral solution along the needle track.

### 2.6. Behavioral Tests

Modified neurological severity score (mNSS): Neurological function was measured using the mNSS both at baseline (at post TBI, prior to treatment) and after treatment. The total score ranges from 0 (normal) to 18 (maximal deficit) [33]. According to established criteria, scores of ≤6, 7–12, and ≥13 correspond to mild, moderate, and severe injury, respectively. Only animals with a moderate injury severity (mNSS 7–12 at baseline) were included in the study. All animals meeting the a priori inclusion criteria—i.e., mNSS score within the designated range at baseline and survival through the experimental endpoint—were included in the final analysis, with no exclusions of animals, experimental units, or data points in any of the groups.

Rotarod Test: Motor coordination and balance were assessed using an accelerating rotarod apparatus. An adaptive training phase was conducted 24 h prior to formal testing. During each trial, the rod accelerated linearly from 5 to 40 rpm over a 5 min period. The latency to fall (seconds) and the maximum rotarod speed achieved at the time of falling were recorded for each animal [34]. Three consecutive trials were performed on post-injury day 3 and the mean values were calculated for subsequent analysis.

Novel Object Recognition Test (NORT): Recognition memory was evaluated using the NORT paradigm, which consisted of three consecutive phases. Prior to the training phase, each mouse was placed in an empty open-field arena and allowed to explore freely for 5 min to acclimate to the testing apparatus. Subsequently, the training phase was conducted, in which two identical objects were placed symmetrically in the arena, and the animal was permitted to explore for 5 min. In the test phase, performed 24 h after training, one of the familiar objects was replaced with a novel object, and exploratory behavior was recorded over a 5 min session [35]. Throughout the test phase, we recorded both locomotor activity (total distance traveled and average speed) and object-directed exploration (frequency and duration of exploration directed toward the novel vs. familiar object). To control for olfactory cues, objects and arenas were thoroughly cleaned with 70% ethanol and allowed to dry completely between trials.

Y-Maze Test: Spatial working memory and exploratory behavior were examined using a Y-maze apparatus (three arms, each 35 cm in length, with an angle of 120° between arms). The test comprised two phases. In the training phase, one arm (designated as the novel arm) was blocked by a removable opaque partition, and mice were allowed to explore the two open arms (arms B and C) for 10 min. After a 1 h inter-phase interval, the test phase was conducted with all three arms accessible, and each mouse was allowed to freely explore the maze for 10 min [35]. The percentage of entries into and time spent in the novel arm relative to the total entries and total time were calculated as indices of novelty-preference memory.

Rotarod, NOR, Y-maze were performed on post-injury day 3 following the final EA session, and all tests were conducted by investigators blinded to group allocation.

### 2.7. Tissue Collection

Tissue samples for Western blot, ELISA, and immunofluorescence were collected immediately after behavioral testing on post-injury day 3. At the end of the behavioral tests, mice were deeply anesthetized and decapitated. Brains were rapidly removed, and cortical tissues surrounding the lesion site were dissected on ice. The collected tissues were immediately frozen in liquid nitrogen and stored at −80 °C for subsequent protein and molecular analyses. For histological examination, separate cohorts of mice were transcardially perfused with 0.9% saline followed by 4% paraformaldehyde, and brain sections containing the peri-lesional cortex were prepared.

### 2.8. Transmission Electron Microscopy (TEM)

Fresh cortical tissue samples were trimmed into blocks of no more than 1 mm × 1 mm × 1 mm and immediately immersed in electron microscopy fixative at 4 °C for 4 h. Following primary fixation, specimens were rinsed three times with 0.1 M phosphate-buffered saline (PBS, pH 7.4), 15 min per wash. For post-fixation, samples were incubated in 1% osmium tetroxide (OsO_4_) prepared in 0.1 M PBS (pH 7.4) at room temperature (20 °C) for 2 h, and then washed again with PBS three times (15 min each). Dehydration was carried out through an ascending ethanol series of 50%, 70%, 80%, 90%, 95%, and 100% (twice), with 15 min incubation per step. Subsequently, specimens were infiltrated with a 1:1 mixture of acetone and 812 embedding resin overnight, followed by pure 812 resin overnight, and finally embedded in resin blocks that were polymerized at 60 °C for 48 h. Ultrathin sections (60–80 nm in thickness) were cut using an ultramicrotome, mounted onto copper grids, and subjected to uranium-lead double staining (2% uranyl acetate saturated aqueous solution and lead citrate, 15 min each). Grids were then air-dried overnight at room temperature. Ultrastructural examination was performed using a transmission electron microscope (Tecnai G2 20 TWIN, FEI, Hillsboro, OR, USA) operated at an accelerating voltage of 200 kV. From the acquired TEM micrographs, synaptic density, synaptic morphology (postsynaptic density thickness and curvature), and mitochondrial ultrastructure were quantified.

### 2.9. Double Immunofluorescence Labeling for p-Drp1 Ser616 and TOM20

Following antigen retrieval and blocking, sections were incubated overnight at 4 °C with a rabbit anti-phospho-Drp1 (Ser616) polyclonal antibody (1:200, AF8470, Affinity Biosciences, Cincinnati, OH, USA) and Anti-TOM20 antibody (1:200, F0513, Selleck Chemicals, Houston, TX, USA) in combination with a mouse monoclonal antibody against the target protein of interest. After three washes with PBS, the sections were incubated with the corresponding secondary antibodies—Alexa Fluor^®^ 488-conjugated goat anti-rabbit IgG (1:500, Invitrogen, Carlsbad, CA, USA) and Alexa Fluor^®^ 594-conjugated goat anti-mouse IgG (1:500, Invitrogen, Carlsbad, CA, USA)—for 1 h at room temperature in the dark. Nuclei were counterstained with DAPI (1:1000, Sigma Aldrich/Merck, Darmstadt, Germany) for 10 min. After a final wash, the sections were mounted with anti-fade fluorescent mounting medium and covered with coverslips. Fluorescence images were captured using a confocal laser scanning microscope under appropriate excitation wavelengths.

### 2.10. Patch-Clamp Recording

Brain slices containing the ipsilesional cortex were prepared for whole-cell patch-clamp recordings. Briefly, mice were decapitated, and the brains were rapidly removed and immersed in ice-cold artificial cerebrospinal fluid (ACSF) continuously oxygenated with carbogen (95% O_2_/5% CO_2_). Coronal slices of 350 μm thickness were sectioned using a vibrating microtome (VT1200S, Leica Biosystems, Nussloch, Germany). Following sectioning, slices were allowed to recover in ACSF at 34 °C for 30 min and then kept at room temperature (22–25 °C) for at least 1 h prior to recording. All recordings were completed within 6 h after recovery.

Whole-cell patch-clamp recordings were performed to measure spontaneous inhibitory postsynaptic currents (sIPSCs) from neurons located in the perilesional cortex. Recording electrodes (3–5 MΩ when filled with internal solution) were pulled from borosilicate glass capillaries. For measurement of spontaneous inhibitory postsynaptic currents (sIPSCs), the recording electrodes were backfilled with an intracellular solution consisting of (in mM): 125 CsCH_3_SO_3_, 5 CsCl, 10 phosphocreatine, 10 HEPES, 0.2 EGTA, 1 MgCl_2_, 4 Mg-ATP, 0.3 Na-GTP, and 5 QX-314 (pH 7.30, 280 mOsm) [36]. Whole-cell voltage-clamp recordings were conducted at a holding potential at +10 mV for sIPSCs.

Signals were amplified using a MultiClamp 700B amplifier (Molecular Devices, LLC, San Jose, CA, USA), digitized with a Digidata 1440A converter, and acquired via pCLAMP software (version 10.7, Molecular Devices, LLC, San Jose, CA, USA). Cells with a resting membrane potential negative to −50 mV, stable series resistance (<20 MΩ, with <20% change during recording), and minimal leak current were included for analysis. All recordings were performed at room temperature (22–25 °C). Data were filtered at 2 kHz and digitized at 10 kHz.

### 2.11. JC-1 Staining for Mitochondrial Membrane Potential

Mitochondrial membrane potential (ΔΨm) was evaluated using the JC-1 fluorescent probe. Briefly, single-cell suspensions were incubated with JC-1 working solution at 37 °C in a 5% CO_2_ atmosphere for 20 min in the dark. After incubation, cells were pelleted by centrifugation, and the supernatant was discarded. The pellet was then washed three times with ice-cold 1× assay buffer and finally resuspended in the same buffer for flow cytometric analysis. Fluorescence was detected using appropriate excitation/emission settings (excitation 488 nm; JC-1 monomers detected at emission 530 nm, and aggregates at emission 590 nm). The mitochondrial membrane potential was expressed as the fluorescence intensity ratio of JC-1 aggregates (red, 590 nm) to JC-1 monomers (green, 530 nm), with a decreased ratio indicating depolarization of the mitochondrial membrane.

### 2.12. AMP and ATP Measurements

Adenosine monophosphate (AMP) and adenosine triphosphate (ATP) levels in cortical tissue homogenates were measured using commercial colorimetric assay kits, following the manufacturers’ recommended protocols. Specifically, AMP concentration was determined using a Mouse AMP ELISA Kit (BES0019K, Shanghai BioESN Biotechnology Co., Ltd., Shanghai, China), while ATP content was quantified using an ATP Content Assay Kit (Beijing Boxbio Science & Technology Co., Ltd., Beijing, China). Absorbance readings were acquired on a microplate reader, with optical density measured at 450 nm for the AMP assay and at 340 nm for the ATP assay. All samples were run in duplicate, and the final concentrations were calculated against standard curves generated from serially diluted reference standards provided with each kit.

### 2.13. Quantitative Real-Time PCR (qPCR)

Total RNA was extracted from cortical tissue samples using a commercial RNA isolation kit (Guangzhou Songsheng Biotechnology Co., Ltd., Guangzhou, China). RNA concentration and purity were assessed spectrophotometrically by measuring the A_260_/A_280_ ratio, with only samples falling within the range of 1.8–2.0 processed further. One microgram of total RNA from each sample was reverse-transcribed into complementary DNA (cDNA) using a reverse transcription kit (Guangzhou Songsheng Biotechnology Co., Ltd., Guangzhou, China) following the manufacturer’s protocol. Quantitative real-time PCR (qRT-PCR) was subsequently performed on a real-time PCR detection system using SYBR Green Master Mix (Guangzhou Songsheng Biotechnology Co., Ltd., Guangzhou, China). Each reaction was run in triplicate, and no-template controls (NTC) were included in every run to exclude potential contamination or non-specific amplification.

Gene-specific primer pairs for CaMKII and the internal reference gene β-actin were designed based on previously published sequences and synthesized by Sangon Biotech Co., Ltd., Shanghai, China. Primer specificity was verified by melting-curve analysis, which revealed a single peak for each primer pair, indicating no detectable primer-dimer formation or off-target amplification. Amplification efficiency was assessed using standard curves from serially diluted pooled cDNA. All primer pairs exhibited amplification efficiencies within the acceptable range of 90–110%, with correlation coefficients (R^2^) exceeding 0.99. Relative expression levels of CaMKII were calculated using the 2^−ΔΔCt^ method, with β-actin serving as the endogenous reference gene.

The primer sequences were as follows [37,38]:

CaMKII: forward 5′-TATCCGCATCACTCAGTACCTG-3′, reverse 5′-GAACTGGACGATCTGCCATTT-3′

β-actin: forward 5′-AGTGTGACGTTGACATCCGT-3′, reverse 5′-TGCTAGGAGCCAGAGCAGTA-3′.

### 2.14. Western Blot (WB)

Total protein was extracted from the brain tissue using RIPA lysis buffer containing protease and phosphatase inhibitors. Protein concentrations were determined with a BCA assay kit. Equal amounts of protein were separated by SDS-PAGE and transferred onto PVDF membranes. After blocking with 5% non-fat milk, the membranes were incubated overnight at 4 °C with primary antibodies against CaMKII (1:1000, MA1-048, Invitrogen), Drp1 (phospho-Ser616) (1:1000, AF8470, Affinity Biosciences), Drp1 (1:5000, 26187-1-AP, Wuhan Sanying Biotechnology Co., Ltd., Wuhan, China), MFN2 (1:1000, HA720073, HUABIO, Hangzhou HuaAn Biotechnology Co., Ltd., Hangzhou, China), PSD95 (1:1000, ab18258, Abcam Ltd., Cambridge, UK), SYN (1:1000, ab8049, Abcam), and β-actin (1:1000, #4970, Cell Signaling Technology, Inc., Danvers, MA, USA). Following incubation with appropriate HRP-conjugate secondary antibodies (1:2000, Anti-mouse IgG, #7076 and 1:2000, Anti-rabbit IgG, #7074, Cell Signaling Technology), protein bands were visualized using an enhanced chemiluminescence (ECL) substrate. Protein blot intensities were quantified using ImageJ software (version 1.52a, National Institutes of Health, Bethesda, MD, USA).

### 2.15. ELISA Quantification of Immunoprecipitated Drp1

Total protein from tissue lysates (200 μg per group) was subjected to IP using 2 μg of anti-Drp1 antibody and 50 μL of Protein A/G agarose beads overnight at 4 °C. After washing three times with ice-cold IP lysis buffer (normal rabbit IgG as negative control), bound proteins were eluted with 100 μL of 0.1 M glycine-HCl (pH 2.8) for 10 min at room temperature and neutralized with 1/10 volume of 1 M Tris-HCl (pH 8.0). A blank control (10 μL eluate + 1 μL neutralization buffer) was prepared for background correction.

Drp1 in the eluates was quantified by sandwich ELISA. Samples were 5-fold diluted (10 μLeluate + 40 μL diluent) and assayed in duplicate alongside standards, blanks, and background controls. After sequential incubations with HRP-conjugated detection antibody and chromogenic substrates, absorbance was read at 450 nm. A standard curve (R^2^ ≥ 0.99) was constructed, and sample concentrations were calculated after subtracting background and IgG controls.

### 2.16. Statistical Analysis

All exclusions were performed before randomization; therefore, data from excluded animals were not included in the subsequent statistical analyses. The order of treatments and measurements was alternated across groups, cage locations were randomly assigned, and blinding was applied during outcome assessment. All data are presented as mean ± standard error of the mean (SEM). Statistical analyses were performed using SPSS 25.0 software. Normality was assessed by the Shapiro Wilk test, and homogeneity of variances was assessed by Levene’s test. Comparisons among multiple groups were performed using one-way analysis of variance (ANOVA), followed by Tukey HSD post hoc tests for pairwise comparisons when the main effect was significant. A *p*-value of less than 0.05 was considered statistically significant. Sample sizes were chosen to provide adequate statistical power to detect biologically relevant differences while adhering to the 3R principles.

## 3. Results

### 3.1. EA Alleviates Motor and Cognitive Deficits in TBI Mice

**Figure 1:**
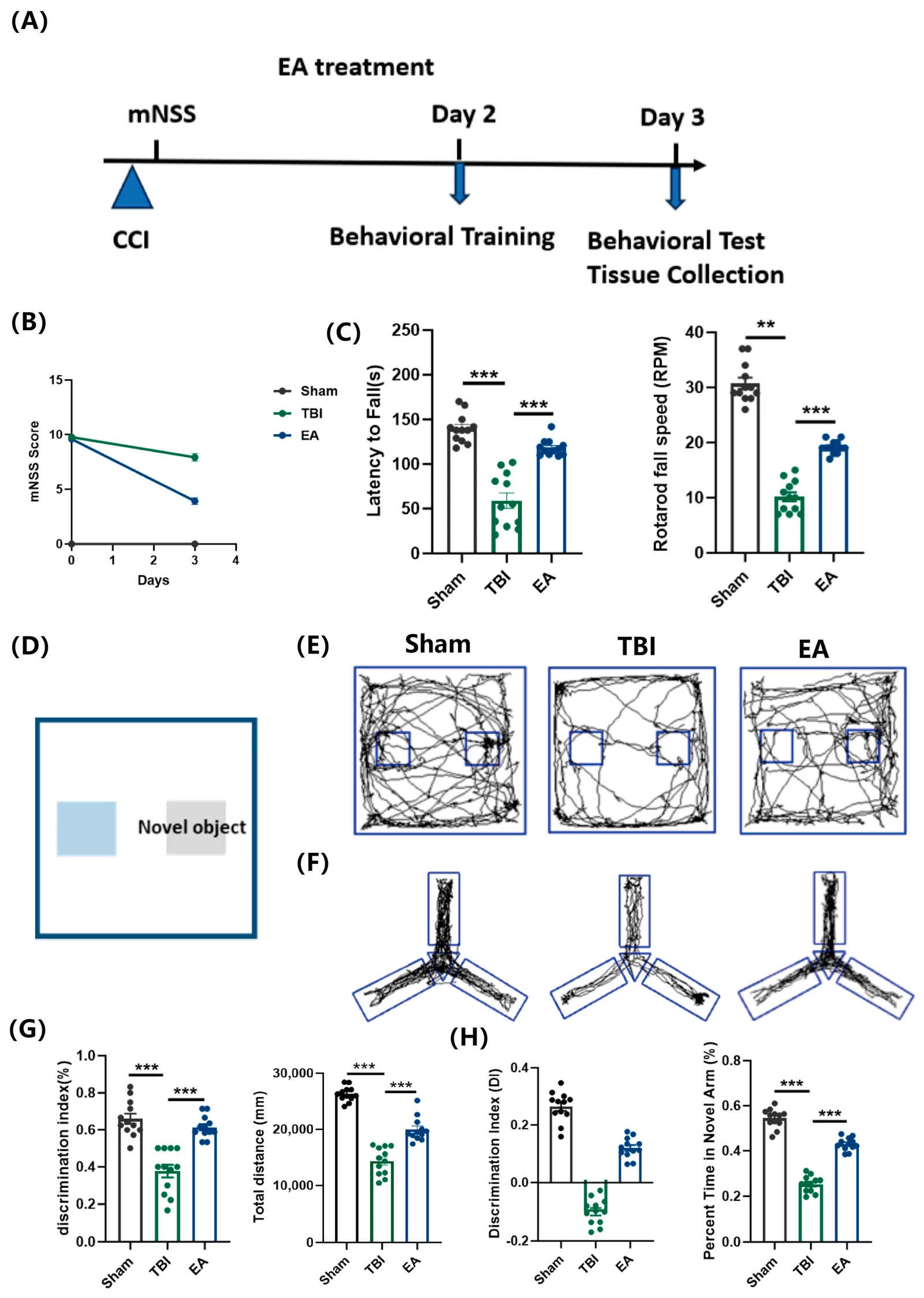
EA improves neurological deficits in TBI mice. (**A**) Schematic timeline of Animal Experiment 1. (**B**) Assessment of neurological deficits by mNSS. (**C**) Assessment of motor function by the accelerating rotarod test. (**D**) Graphical abstract of the NORT experimental procedure. (**E**) Representative movement tracks of mice during the test session of NORT. (**F**) Representative movement tracks of mice during the test session of Y maze. (**G**) Quantitative analysis of exploratory behavior and cognitive performance in the NORT. (**H**) Quantitative analysis of cognitive performance in the Y maze test. *n* = 12 mice per group, ** *p* < 0.01, *** *p* < 0.001.

The successful induction of moderate TBI was confirmed by modified Neurological Severity Scores (mNSS) ranging from 7 to 12 (Figure 1B). Consistent with the neurological deficits, TBI mice exhibited significant motor coordination impairments in the rotarod test, marked by decreased latency to fall and reduced tolerance to acceleration; EA treatment effectively reversed these impairments, significantly increasing both parameters (Figure 1C). In the novel object recognition test (NORT), TBI mice showed a decreased preference for the novel object, indicating impaired recognition memory. In addition, consistent with the rotarod results, TBI mice showed reduced locomotor activity (total distance) during the test session (Figure 1E,G). Notably, EA treatment significantly ameliorated these deficits compared to the TBI group, effectively restoring both locomotion and novel object preference (Figure 1E,G). In the Y-maze test, TBI mice displayed impaired spatial recognition memory, as shown by reduced exploration of the novel arm. EA treatment significantly ameliorated this deficit, increasing novel arm exploration time (Figure 1F,H). Collectively, these behavioral results demonstrate that EA promotes functional recovery after TBI by alleviating motor and cognitive deficits.

### 3.2. EA Promotes Functional Recovery Through Attenuation of Synaptic Loss Following TBI

**Figure 2:**
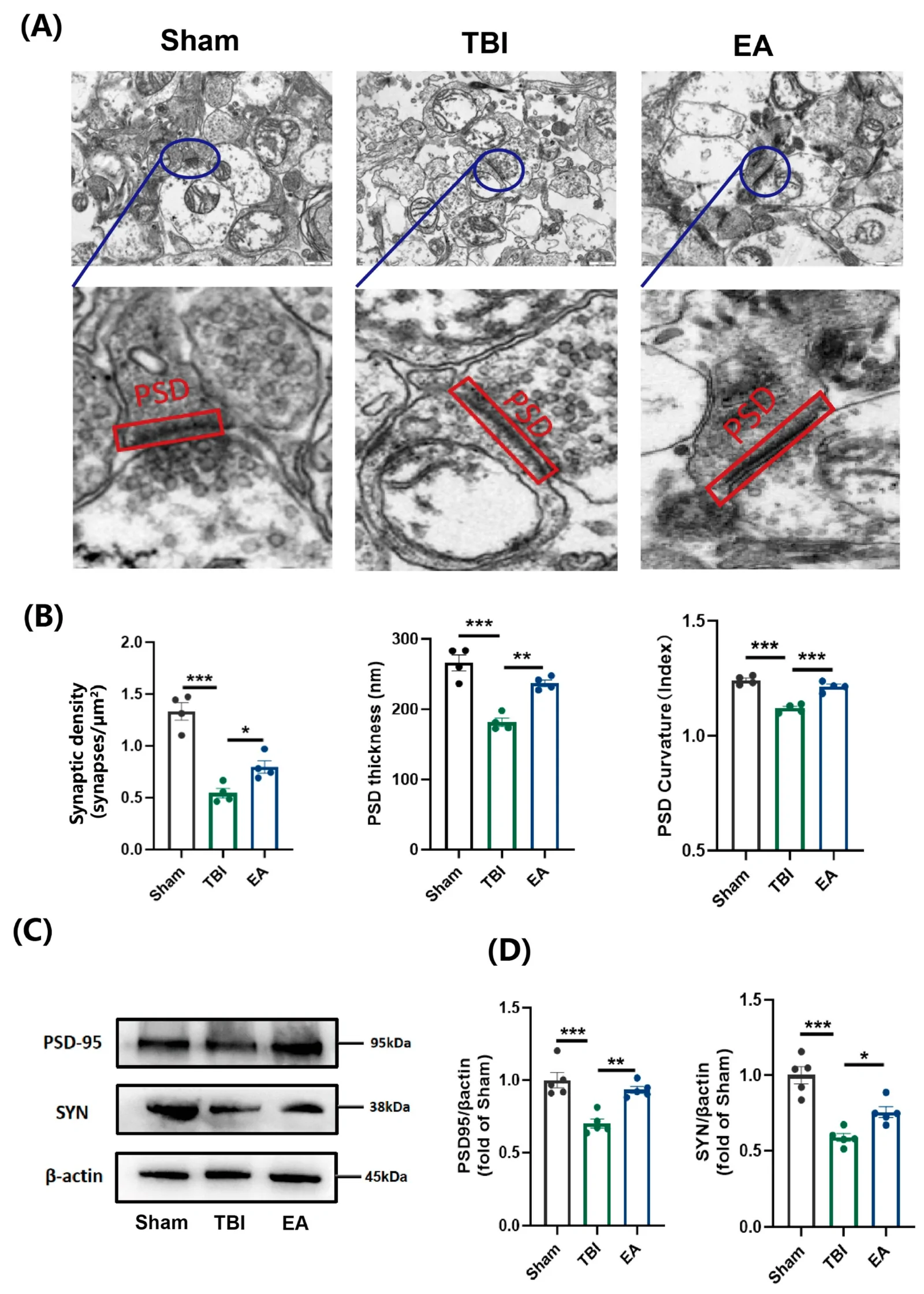
EA treatment mitigated TBI-induced synaptic loss in the ipsilesional cortex. (**A**) The synaptic ultrastructure was examined and analyzed by TEM. (**B**) The quantitative analysis of TEM. (Scale bar = 500 nm; n = 4 mice per group.) (**C**,**D**) The protein levels of the synaptic-related markers PSD95 and SYN were quantitatively determined by WB. (*n* = 5 mice per group). All data met the assumptions of normality (Shapiro–Wilk) and homogeneity of variances (Levene’s test). * *p* < 0.05, ** *p* < 0.01, *** *p* < 0.001. The original Western blot images can be found in the Supplementary Materials.

Given that synaptic loss is a critical pathological hallmark underlying neurological dysfunction after TBI [33], we hypothesized that EA exerts neuroprotective effects by attenuating this process. Corroborating this hypothesis, TEM ultrastructural analysis demonstrated a significant decrease in synaptic density in the ipsilesional cortex of TBI mice. Furthermore, the remaining synapses showed clear structural deterioration, including a thinner and less curved postsynaptic density relative to sham-operated animals (Figure 2A). Conversely, EA treatment demonstrated a significant rescue effect on synaptic ultrastructure. Treated mice exhibited restored synaptic density and improved PSD morphology (increased thickness and curvature) compared to the TBI group (Figure 2B). Consistent with the ultrastructural findings, WB analysis revealed that TBI significantly downregulated the expression of synaptic markers PSD95 and SYN, whereas EA treatment effectively restored their protein levels (Figure 2C,D and Figure S1). Collectively, these results demonstrate that EA ameliorates TBI-induced damage to both the morphology and function of synapses. The repair of synaptic integrity is thus identified as a potential mechanism contributing to EA-promoted neurological recovery after TBI.

### 3.3. EA Promotes Energy Supply and Preserves Mitochondrial Structure and Function

**Figure 3:**
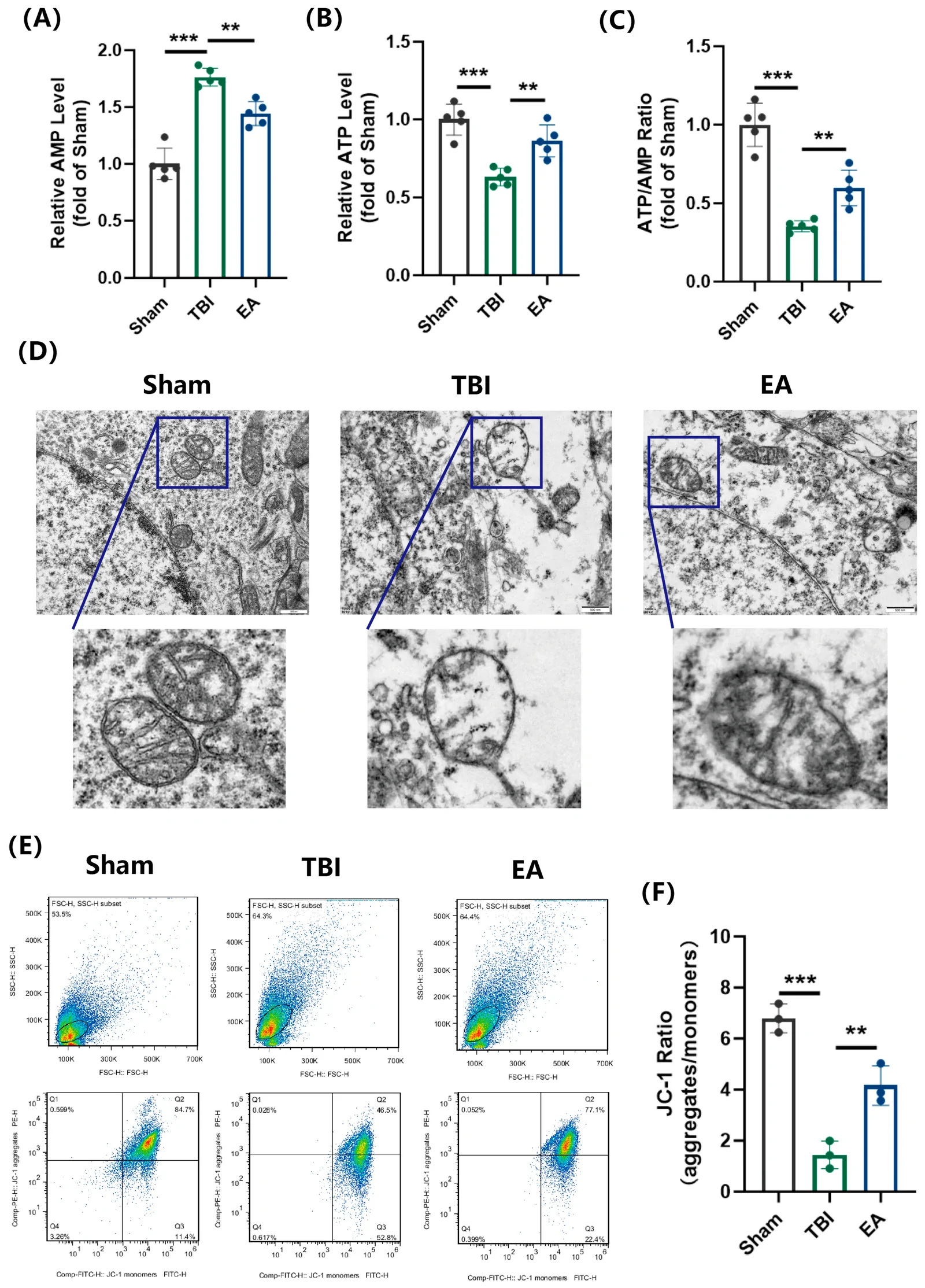
EA treatment rescued mitochondrial ultrastructure and function in the ipsilesional cortex of TBI mice. (**A**–**C**) The concentrations of ATP and AMP were measured by biochemical assays (*n* = 5 mice per group). (**D**) Observation of the ultrastructure of mitochondria by TEM (Scale bar = 500 nm). (**E**,**F**) Mitochondrial membrane potential was assessed by JC-1 staining and flow cytometry, JC-1 aggregates (red) and JC-1 monomers (green) were quantified (*n* = 3 mice per group). All data met the assumptions of normality (Shapiro–Wilk) and homogeneity of variances (Levene’s test). ** *p* < 0.01, *** *p* < 0.001.

To comprehensively evaluate mitochondrial status, we employed TEM for ultrastructure, flow cytometry for function, and ELISA for energy metabolism. ELISA revealed a profound energy deficit in TBI mice, characterized by decreased ATP and increased AMP levels, which was effectively reversed by EA treatment through the normalization of ATP production (Figure 3A–C).

TEM analysis showed that TBI induced severe mitochondrial ultrastructural damage, including swelling, vacuolization, and cristae loss, which was substantially rescued by EA treatment (Figure 3D). This structural restoration contributed to the recovery of mitochondrial membrane potential, as evidenced by an increased JC-1 aggregate/monomer ratio following EA intervention (Figure 3E,F). Collectively, these results indicate that EA enhances energy metabolism after TBI by repairing mitochondrial integrity and function, a mechanism underlying its efficacy in attenuating synaptic loss.

### 3.4. EA Attenuates TBI-Induced Mitochondrial Dysfunction by Counteracting Excessive Fission

**Figure 4:**
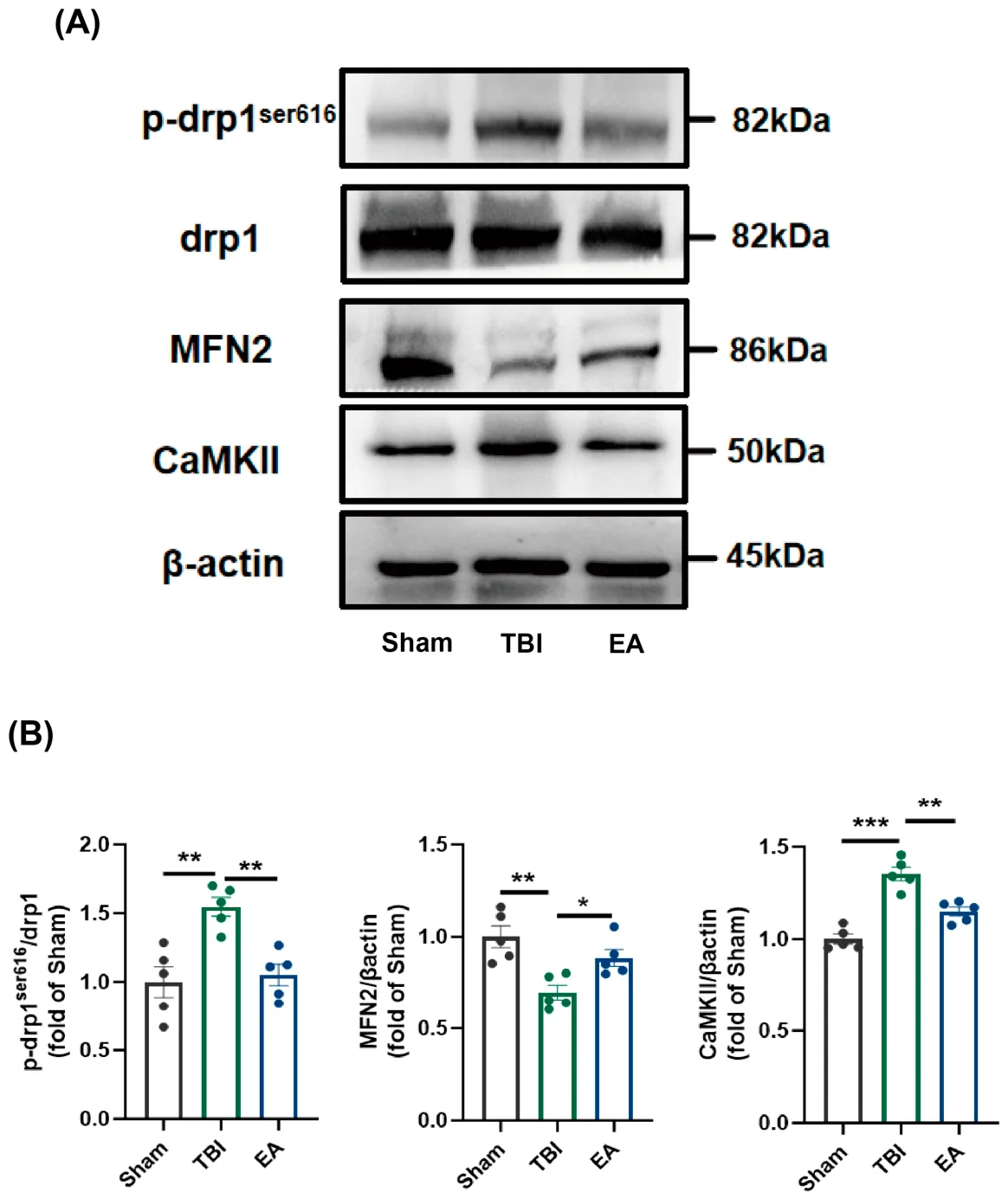
EA modulates mitochondrial dynamics-related proteins in the ipsilesional cortex of TBI mice (**A**,**B**) WB analysis of p-Drp1, Drp1, MFN2, and CaMKII protein levels in the ipsilesional cortex of TBI mice. (*n* = 5 mice per group). * *p* < 0.05, ** *p* < 0.01, *** *p* < 0.001. The original Western blot images can be found in the Supplementary Materials.

WB analysis of mitochondrial dynamics-related proteins revealed that TBI significantly increased the p-Drp1 (Ser616)/Drp1 ratio while concurrently decreasing MFN2 levels, indicating an imbalance in mitochondrial dynamics skewed toward excessive fission (Figure 4A,B and Figure S1). In contrast, EA treatment effectively corrected the TBI-induced shift by reducing the p-Drp1 (Ser616)/Drp1 ratio and upregulating MFN2 expression (Figure 4A,B and Figure S1). Furthermore, CaMKII expression was markedly elevated after TBI, and this increase was reversed by EA treatment (Figure 4A,B and Figure S1). These results suggest that CaMKII may serve as a key mediator of the neuroprotective effects elicited by EA.

Specifically, EA attenuated pathological mitochondrial fission through a dual effect: suppressing Drp1 phosphorylation at Ser616 and enhancing mitochondrial fusion activity, thereby counteracting the TBI-induced shift in mitochondrial dynamics toward excessive fission. These findings indicate that EA mitigates TBI-induced mitochondrial fission via the CaMKII/Drp1 signaling axis: EA inhibits TBI-induced CaMKII overexpression, leading to reduced Drp1 phosphorylation at Ser616 and consequently suppressing excessive mitochondrial fission.

### 3.5. EA Mitigates Post-TBI Mitochondrial Fission by Targeting the CaMKII/Drp1 Signaling Axis

**Figure 5:**
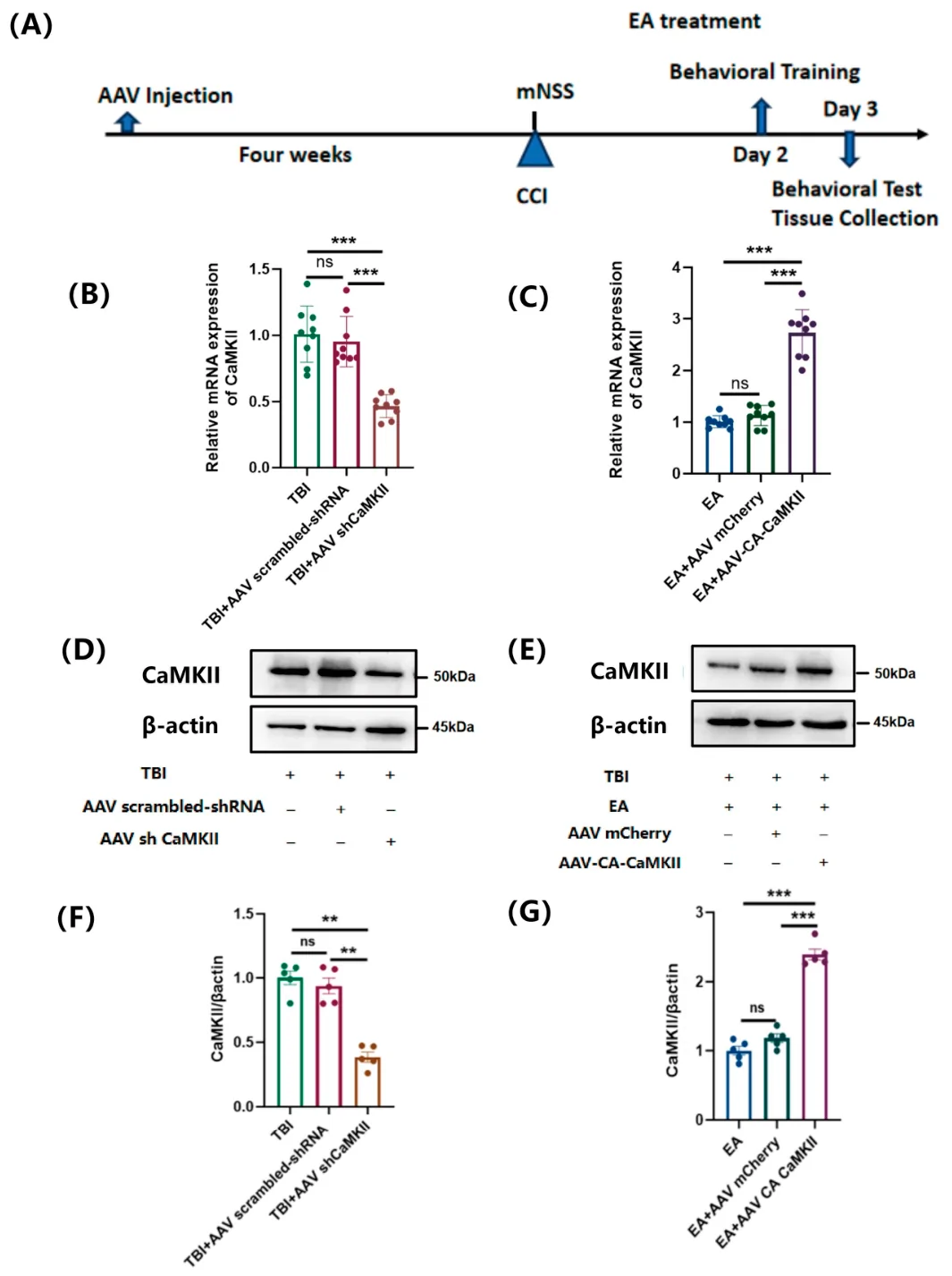
Verification of CaMKII knockdown/overexpression by AAV. (**A**) Schematic timelines of Animal Experiment 2 and Animal Experiment 3. (**B**,**C**) mRNA expression levels of CaMKII as determined by qPCR. (*n* = 9 mice per group). (**D**–**G**) WB analysis of CaMKII protein levels in the ipsilesional cortex of TBI mice (*n* = 5 mice per group). ** *p* < 0.01, *** *p* < 0.001; ns, not significant (*p* ≥ 0.05). The original Western blot images can be found in the Supplementary Materials.

To determine whether CaMKII plays a causal role in the therapeutic effects of EA, we used AAV-mediated approaches to knockdown or overexpress CaMKII via intracerebroventricular injection in TBI mice (Figure 5A). Successful transfection was confirmed by significant alterations in CaMKII mRNA and protein levels compared to the blank vector control (Figure 5B–G, Figures S1 and S2). CaMKII knockdown (AAV-shCaMKII) was found to significantly reduce CaMKII expression in the ipsilesional cortex relative to the TBI group and AAV-scrambled shRNA controls (Figure 5B,D,F, Figures S1 and S2), whereas AAV-CA-CaMKII increased CaMKII expression relative to the EA and AAV-mCherry controls (Figure 5C,E,G, Figures S1 and S2). These functional rescue experiments provide direct evidence that CaMKII is a key mediator of EA’s neuroprotective effects.

**Figure 6:**
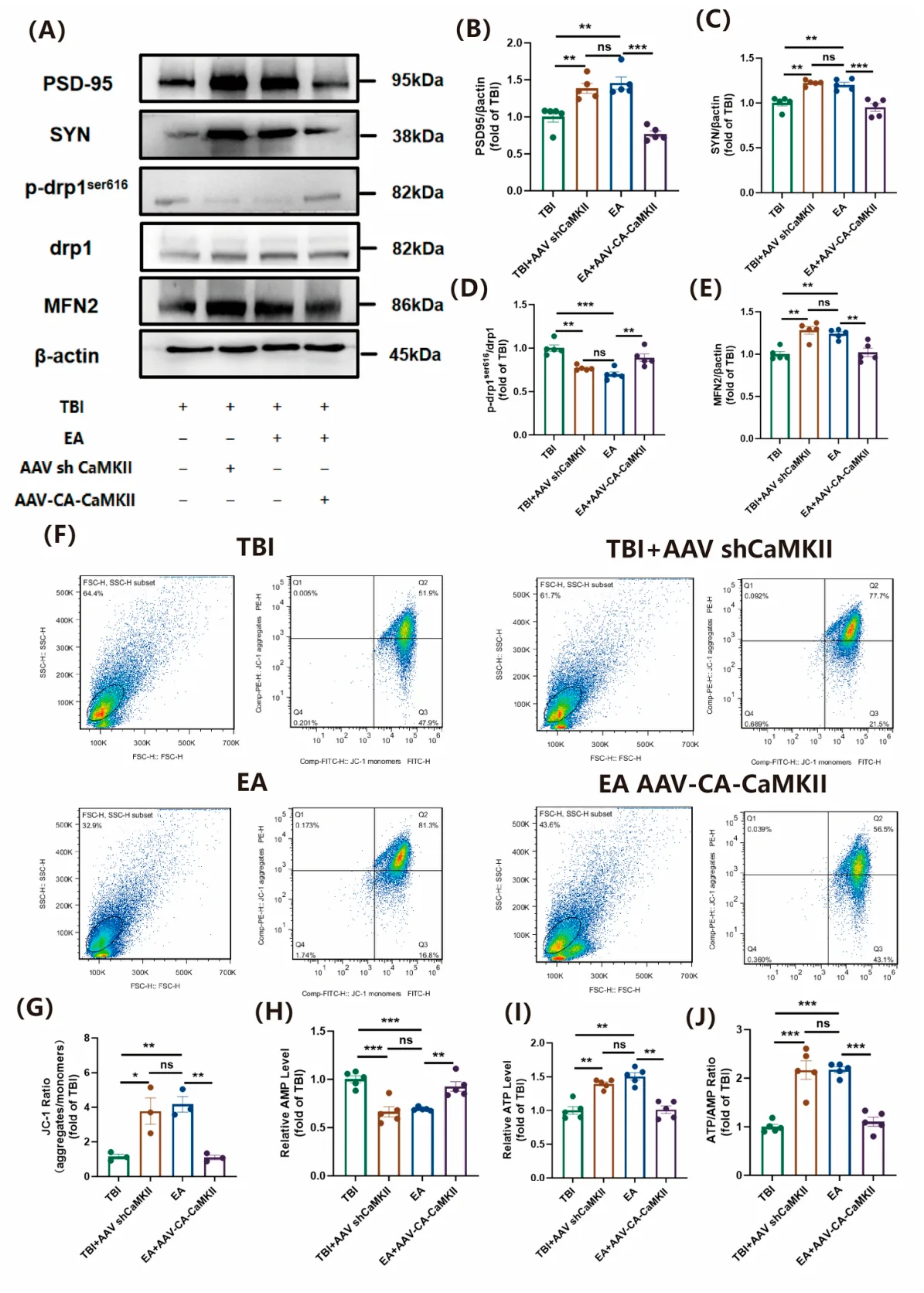
CaMKII is a key target of EA in reducing mitochondrial fission and promoting mitochondrial functional recovery after TBI. (**A**–**E**) WB analysis of p-Drp1, Drp1, MFN2, PSD95 and SYN protein levels in the ipsilesional cortex of TBI mice (*n* = 5 mice per group); (**F**,**G**) Mitochondrial membrane potential was assessed by JC-1 staining and flow cytometry, JC-1 aggregates (red) and JC-1 monomers (green) were quantified, (*n* = 3 mice per group); (**H**–**J**) The concentrations of ATP and AMP were measured by biochemical assays. (*n* = 5 mice per group). All data met the assumptions of normality (Shapiro–Wilk) and homogeneity of variances (Levene’s test). * *p* < 0.05, ** *p* < 0.01, *** *p* < 0.001; ns, not significant (*p ≥* 0.05). The original Western blot images can be found in the Supplementary Materials.

We examined whether EA regulates mitochondrial fission via the CaMKII/Drp1 pathway to alleviate TBI-induced mitochondrial dysfunction. WB analysis showed that EA upregulated the synaptic markers PSD95 and SYN, an effect mimicked by CaMKII knockdown and abolished by CaMKII overexpression (Figure 6A–C and Figure S1). EA also reduced Drp1 phosphorylation at Ser616, a step in its activation. CaMKII knockdown achieved this effect, whereas CaMKII overexpression blocked EA’s suppression of Drp1 activation (Figure 6A,D and Figure S1). Conversely, CaMKII overexpression offset the promoting effect of EA on Mfn2 (Figure 6A,E and Figure S1). We then assessed mitochondrial membrane potential (ΔΨm) using the JC-1 aggregate/monomer ratio. EA restored the depolarized ΔΨm in the ipsilesional cortex after TBI (Figure 6F,G). CaMKII knockdown mimicked EA’s effect, while CaMKII overexpression blocked EA’s restoration of ΔΨm (Figure 6F,G). ELISA confirmed that EA increased ATP levels in the ipsilesional cortex. The AAV-shCaMKII group showed a similar increase, whereas constitutive CaMKII activation in EA-treated mice blocked this improvement (Figure 6H–J). Together, these results indicate that the CaMKII/Drp1 pathway mediates EA’s effects on mitochondrial function after TBI.

### 3.6. CaMKII/Drp1 Pathway Is Involved in EA-Induced Suppression of Mitochondrial Fission After TBI

**Figure 7:**
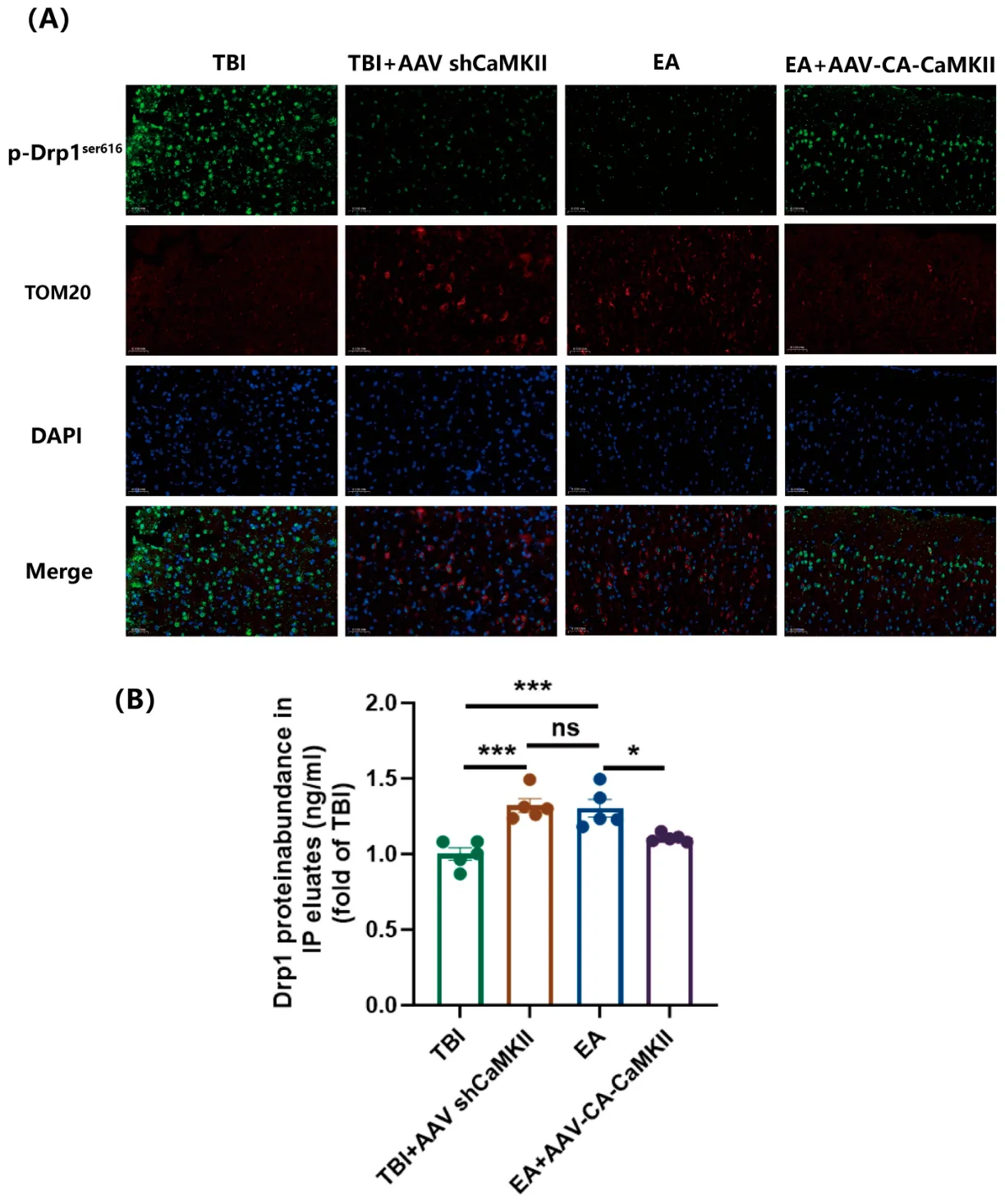
CaMKII/Drp1 axis mediates EA-induced inhibition of mitochondrial fission. (**A**) Representative immunofluorescence images of TOM20 (red), p-Drp1(Ser616) (green), and DAPI (blue) in the peri-contusional cortex. Scale bars: 0.05 mm. (**B**) Drp1 protein levels measured by IP-ELISA in cortical lysates. (n = 5 mice per group). * *p* < 0.05, *** *p* < 0.001; ns, not significant (*p* ≥ 0.05).

To further validate the involvement of the CaMKII/Drp1 axis in EA-mediated inhibition of mitochondrial fission, we examined the expression of p-Drp1 (Ser616), the mitochondrial marker TOM20, and total Drp1 protein in pericontusional cortical tissues. Immunofluorescence analysis revealed that p-Drp1 (Ser616) expression was markedly lower in the EA group than in the TBI group, whereas TOM20 levels were significantly higher in the EA group than in the TBI group (Figure 7A). Consistent with these findings, CaMKII knockdown (AAV-shCaMKII) recapitulated the effects of EA on both p-Drp1 (Ser616) and TOM20 expression. Conversely, constitutively active CaMKII overexpression (AAV-CA-CaMKII) abolished the EA-induced changes, leading to increased p-Drp1 (Ser616) and decreased TOM20 levels (Figure 7A).

In parallel, Drp1 was immunoprecipitated from cortical lysates and its protein levels were quantified by ELISA. As shown in Figure 7B, EA intervention further increased Drp1 protein expression in TBI mice, suggesting a compensatory upregulation of total Drp1 levels in response to reduced phosphorylation-dependent mitochondrial translocation. These findings indicate that EA not only suppresses Drp1 activation (as reflected by decreased p-Drp1^Ser616^/Drp1 ratio) but also modulates its overall protein abundance, further supporting the involvement of Drp1 in EA-mediated suppression of mitochondrial fission.

### 3.7. CaMKII Plays a Pivotal Role in EA-Mediated Improvement of Synaptic Transmission and Neurological Recovery After TBI

**Figure 8:**
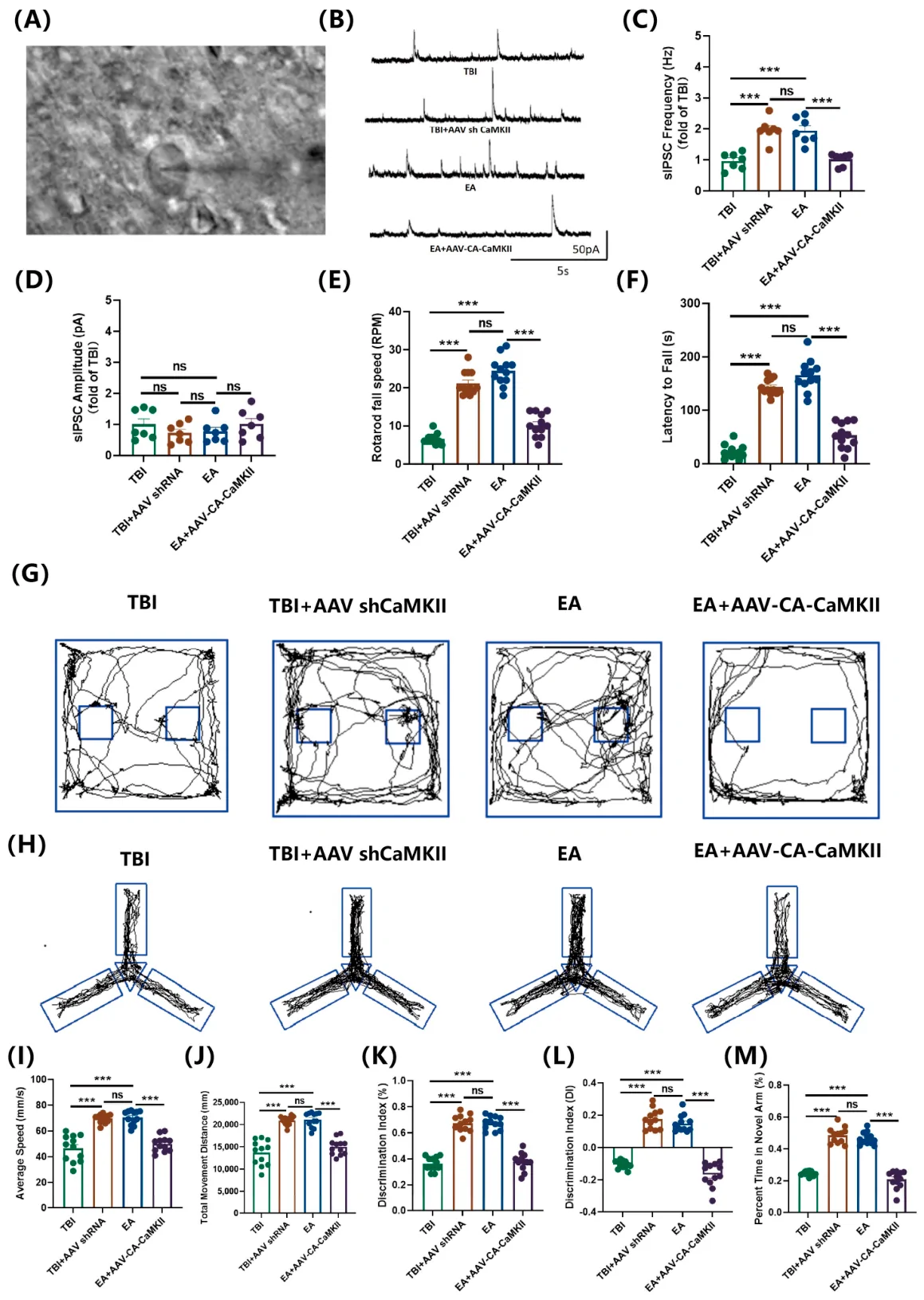
CaMKII is a key target for EA in improving synaptic transmission and neurological function after TBI. (**A**–**D**) Whole-cell patch-clamp recording was used to measure the frequency and amplitude of sIPSC (*n* = 7 brain slices per group); (**E**,**F**) Assessment of motor function by the accelerating rotarod test. (**G**) Representative movement tracks of mice during the test session of NOR. (**H**) Representative movement tracks of mice during the test session of Y maze. (**I**,**J**) Quantitative analysis of exploratory behavior and cognitive performance in the NORT. (**K**–**M**) Quantitative analysis of cognitive performance in the Y maze test. (*n* = 12 mice per group). *** *p* < 0.001; ns, not significant (*p* ≥ 0.05).

To explore whether CaMKII mediates the beneficial effects of EA, we examined synaptic function and behavioral outcomes in TBI mice subjected to different interventions. Patch-clamp recordings showed that EA significantly elevated the frequency—but not the amplitude—of spontaneous inhibitory postsynaptic currents (sIPSCs) (Figure 8A–D). This enhancement was phenocopied by CaMKII knockdown (AAV-shCaMKII) and abolished by constitutively active CaMKII overexpression (AAV-CA-CaMKII) (Figure 8A–D). These findings suggest that EA regulates inhibitory synaptic transmission after TBI in a CaMKII-dependent manner, as indicated by the increased sIPSC frequency following EA treatment.

We next evaluated sensorimotor coordination, exploratory behavior, and spatial memory using the rotarod, novel object recognition (NOR), and Y-maze tests. EA-treated mice showed significantly improved rotarod performance, with longer fall latency and greater tolerance to accelerating speeds relative to TBI controls (Figure 8E,F). This protective effect was recapitulated by CaMKII knockdown and abrogated by CaMKII overexpression (Figure 8E,F). Consistent with these observations, EA also enhanced object exploration and novel arm preference in the NOR and Y-maze tests, and the EA-induced improvement in these behavioral deficits was mimicked by CaMKII knockdown and abolished by CaMKII overexpression (Figure 8G–M). Collectively, these multi-domain behavioral assessments suggest that CaMKII serves as a key mediator of EA-induced neurological recovery following TBI.

## 4. Discussion

TBI remains a leading cause of global mortality and long-term disability, with survivors frequently presenting a constellation of persistent neurological sequelae—including cognitive decline, motor dysfunction, affective disturbances, and impaired consciousness [1,39]. At the pathophysiological level, synaptic loss has been established as a central driver of neurological impairment across a broad spectrum of neurodegenerative conditions. By undermining the fidelity of interneuronal communication, this degenerative process precipitates widespread network dysfunction that ultimately manifests as global brain impairment [40]. Consequently, strategies aimed at preserving or restoring synaptic integrity represent a compelling therapeutic approach, not only for classical neurodegenerative disorders but also for TBI. Although accumulating evidence has documented the beneficial effects of acupuncture in TBI—ranging from mitigation of neuroinflammatory responses and promotion of white matter repair to amelioration of consciousness disturbances [8,41,42]—the precise mechanisms by which acupuncture modulates post-TBI synaptic pathology have remained largely elusive.

In the present study, we demonstrate that electroacupuncture (EA) produces robust improvements in both motor performance and cognitive function in a mouse model of TBI. Focusing our analyses on the ipsilesional cortex, a region that sustains primary mechanical injury and is critically implicated in functional recovery, we observed that these behavioral gains were closely associated with a marked attenuation of synaptic loss. Mechanistically, we provide evidence that EA counteracts TBI-induced aberrant activation of the CaMKII/Drp1 signaling axis, thereby limiting excessive mitochondrial fragmentation and consequently reducing synaptic erosion. To establish causality, we employed AAV-mediated genetic manipulations and found that CaMKII knockdown recapitulated the neuroprotective effects of EA, whereas constitutively active CaMKII overexpression effectively abrogated its therapeutic actions. Collectively, these findings support a model in which EA preserves synaptic architecture after TBI primarily through modulation of the CaMKII/Drp1 pathway and the resultant restoration of mitochondrial homeostasis.

Several rodent models have been established to recapitulate distinct aspects of clinical TBI, including fluid percussion injury, controlled cortical impact (CCI), weight-drop, and blast-induced injury [43]. Among these, the CCI paradigm is widely favored for its translational relevance, as it closely mimics the biomechanical features of human closed-head injury and demonstrates good cross-species applicability [44,45]; we therefore adopted this model in the present study to induce focal cortical contusion. To characterize post-traumatic functional deficits, we employed a standardized behavioral test battery. TBI mice exhibited significant motor incoordination, as reflected by shortened latency to fall and reduced tolerance to accelerating rotation in the rotarod test. Concurrently, they displayed pronounced cognitive impairments, showing diminished exploratory preference for novel objects and novel arms in the novel object recognition and Y-maze tasks, respectively—indicative of deficits in recognition memory and spatial working memory. Notably, EA treatment effectively ameliorated both motor and cognitive deficits across all behavioral paradigms.

Accumulating evidence has implicated widespread synaptic loss as a critical pathological substrate underlying post-TBI neurological dysfunction [46]. Within neural circuits, synaptic density and transmission efficacy are fundamental to information processing and storage. Synaptic loss—defined as a decline in the number, structure, or function of presynaptic terminals, synaptic clefts, and postsynaptic densities—is recognized as an early and powerful predictor of cognitive and motor decline in neurodegenerative conditions such as Alzheimer’s, Parkinson’s, and Huntington’s diseases [4,6,47]. Disruption of multiple signaling pathways can converge on synaptic dismantling and neural network desynchronization, ultimately culminating in persistent functional impairment [6,7]. Guided by this framework, we investigated whether synaptic loss similarly contributes to TBI-associated functional deficits. Our results revealed a marked reduction in synaptic density within the ipsilesional cortex of TBI mice, accompanied by decreased expression of the presynaptic marker synaptophysin and the postsynaptic scaffolding protein PSD95. Importantly, EA treatment significantly attenuated these synaptic alterations, suggesting that preservation of synaptic integrity represents a key mechanism underlying EA-mediated neurological recovery.

Synaptic transmission is energetically demanding and relies on the coordinated integration of multiple homeostatic pathways, including energy metabolism, protein homeostasis, post-translational modification, and glial–immune crosstalk. When these processes are perturbed—particularly under conditions of bioenergetic failure—synaptic loss emerges as a convergent endpoint [4,6]. Synapses are enriched with mitochondria, which primarily generate ATP through oxidative phosphorylation, the predominant energy-producing pathway in neurons [48,49]. Consequently, mitochondrial dysfunction has been established as a common pathological denominator across a broad spectrum of central nervous system disorders. In Parkinson’s disease, for instance, inhibition of mitochondrial complex I impairs respiratory function, depleting ATP and sensitizing neurons to oxidative stress—a key driver of dopaminergic degeneration [50]. In Alzheimer’s disease, post-mortem studies have revealed abnormal mitochondrial morphology and bioenergetic deficits, including impaired glucose metabolism and disrupted calcium homeostasis [49]. This pathological signature extends to psychiatric and neuroinflammatory conditions as well: in major depressive disorder, mitochondrial-derived reactive oxygen and nitrogen species activate caspases, which trigger postsynaptic dendritic spine pruning and impair synaptic plasticity [51]; in multiple sclerosis, compromised mitochondrial function in both glia and neurons exacerbates ATP depletion and oxidative phosphorylation deficits, accelerating demyelination and neuronal loss [52]. Given the central role of mitochondrial impairment in neuropathology, we examined its contribution to TBI and its therapeutic responsiveness. Consistent with findings in other disorders, we observed impaired mitochondrial morphology and function—along with reduced ATP synthesis—in the ipsilesional cortex of TBI mice, and these deficits were closely associated with synaptic loss. Notably, EA treatment effectively reversed these mitochondrial abnormalities, restoring ultrastructural integrity and elevating ATP levels. Collectively, our findings indicate that preservation of mitochondrial integrity and bioenergetic function constitutes a key mechanism by which EA mitigates synaptic loss after TBI.

Mitochondria are intrinsically dynamic organelles that continually undergo fission, fusion, and intracellular trafficking to maintain cellular homeostasis and adapt to fluctuating energy demands [53]. This morphological plasticity is governed by the opposing yet coordinated processes of fission and fusion, which regulate ATP production, respiratory efficiency, and calcium signaling [53,54]. Under physiological conditions, fission and fusion exist in a delicate equilibrium, enabling compensatory responses to metabolic challenges [55]. Disruption of this balance, however, is pathogenic: insufficient fission or excessive fusion leads to accumulation of dysfunctional organelles, whereas excessive fission or insufficient fusion results in pathological fragmentation and bioenergetic compromise [18,55]. Human and animal studies have demonstrated that TBI triggers rapid and region-specific bioenergetic disturbances, with mitochondrial dysregulation detectable in the cortex and striatum as early as 30 min post-injury and in the hippocampus by 7 days post-injury [56]. Notably, synaptic mitochondria exhibit greater vulnerability than their non-synaptic counterparts [57,58]. Our findings align with this temporal and regional vulnerability: at 3 days post-injury, the pericontusional cortex displayed clear mitochondrial morphological and functional impairment, which was effectively counteracted by EA treatment.

The fission–fusion balance is orchestrated by a family of dynamin-related GTPases, among which Drp1 serves as the primary executor of mitochondrial fission [55]. Drp1-driven fission is initiated by its translocation from the cytosol to the mitochondrial outer membrane, where it oligomerizes into higher-order assemblies that constrict the organelle in a GTP-dependent manner [54]. Drp1 activity is tightly regulated by post-translational modifications, with phosphorylation being the most extensively characterized [54]. Specifically, phosphorylation at Ser616 promotes Drp1 mitochondrial recruitment and GTPase activity, thereby driving fission and often augmenting reactive oxygen species production [54,59]. In contrast, phosphorylation at Ser637 generally suppresses fission, although the functional implications of this modification remain context-dependent and continue to be actively investigated [54]. Given that aberrant Drp1 phosphorylation—particularly at Ser616—has been recognized as a pathological hallmark of TBI and a critical driver of neurological impairment, targeted modulation of this phosphorylation event holds substantial therapeutic promise [60,61]. In support of this model, we observed a marked increase in Drp1 phosphorylation at Ser616 in the ipsilesional cortex following TBI, an elevation that was effectively reversed by EA treatment. The concurrent amelioration of mitochondrial deficits and reduction in p-Drp1 Ser616 strongly suggests that EA preserves mitochondrial integrity, at least in part, through normalization of pathogenic Drp1 hyperphosphorylation.

The phosphorylation of Drp1 at Ser616 is a major regulatory center controlled by various kinases including calmodulin-dependent protein kinase II (CaMKII), Rho-associated protein kinase (ROCK), PKCδ, cyclin-dependent kinase 1 (CDK1), and ERK1/2 [54]. Calcium ions, functioning as a master second messenger, are critical for neuronal physiology through diverse processes such as energy metabolism, neurotransmitter release, and synaptic transmission. Under pathological conditions, excessive calcium influx can trigger a cascade of harmful events by hyperactivating calcium-dependent signaling molecules and their downstream networks [22]. CaMKII is an essential serine/threonine kinase for calcium homeostasis, synaptic function, learning, and memory [62]. In addition to its physiological functions, sustained CaMKII activation driven by pathological calcium overload has been implicated in diverse pathological conditions, including alcohol-induced neurotoxicity [24]. In this study, we identified CaMKII as the pivotal upstream kinase responsible for Drp1 hyperphosphorylation at Ser616 following TBI. Our study reveals a fundamental mechanism of EA’s therapeutic action: EA suppresses TBI-induced CaMKII overexpression, thereby reducing pathogenic Drp1 phosphorylation at Ser616. This restores mitochondrial integrity, improves synaptic function, and ultimately leads to behavioral recovery. Thus, our findings establish the CaMKII/Drp1 axis as a key therapeutic target for EA in the treatment of TBI. Although CaMKII inhibition has demonstrated therapeutic benefits in promoting ATP production and reducing synaptic loss after TBI, the dual role of CaMKII in neuroprotection versus neurotoxicity should be noted, as complete inhibition may be detrimental. In contrast, electroacupuncture has been established as a multi-target therapeutic approach that acts through multiple molecular pathways, making it a promising and well-tolerated intervention for TBI [8,26,27,28,29]. Nevertheless, CaMKII possesses a range of additional downstream effectors, and their roles merit further exploration.

We acknowledge that the present study did not include an EA-alone control group in healthy animals. While the primary aim was to evaluate the therapeutic effects of EA under TBI conditions, we recognize that the inclusion of such a group would help exclude non-specific physiological effects of EA and further strengthen the attribution of the observed benefits to the interaction between EA and the injury pathology. In fact, previous studies have demonstrated that EA treatment alone in healthy animals confers superior outcomes compared to non-EA controls, suggesting that EA exerts not only therapeutic but also preventive or neuromodulatory effects beyond the context of injury [63,64]. Nevertheless, future studies incorporating EA-alone controls will be valuable to comprehensively delineate the specific contributions of EA to neural repair beyond its general physiological actions.

The present study focused primarily on the injured cortex, with specific emphasis on mitochondrial function and synaptic ultrastructure, and did not systematically examine potential EA-induced changes in the contralateral hemisphere. Given that pathological changes in the contralateral cortex may exhibit a temporal lag relative to the ipsilateral side, with secondary degeneration and compensatory plasticity occurring at later stages, future investigations incorporating bilateral cortical and other brain regions analysis at multiple time points will be valuable to comprehensively delineate the temporal dynamics and mechanisms underlying EA-mediated neuroprotection and functional recovery after TBI.

Biological factors like estrogen and progesterone, along with gender-related variables such as environmental exposure, risk-taking behaviors, and social support, influence TBI outcomes [65]. To isolate the role of mitochondrial fission-mediated synaptic loss in the effect of electroacupuncture on TBI, we used only male mice in this study. Clinical evidence shows sex differences in TBI incidence, symptom presentation, and recovery [44,66]. Future studies should include female animals and examine how sex, hormonal status, and injury mechanisms interact to affect neurobehavioral outcomes after TBI.

## 5. Conclusions

In conclusion, our study demonstrates that EA preserves neurological function post-TBI through a CaMKII/Drp1-dependent mechanism that maintains mitochondrial and synaptic integrity. We have not only confirmed the pivotal role of CaMKII but also identified the specific cascade it regulates. Although this reveals a promising therapeutic target, further investigation is required to dissect the network in greater detail and explore its full clinical potential.

## Data Availability

The original contributions and/or datasets used presented in this study are included in the article/Supplementary Materials. Further inquiries can be directed to the corresponding author(s).

## Supplementary Materials

The following supporting information can be downloaded at https://www.mdpi.com/article/10.3390/biom16081169/s1, Figure S1: Original Western blot images; Figure S2: qPCR validation of gene expression.

## Abbreviations

The following abbreviations are used in this manuscript:

AAV	Adeno-associated virus
AD	Alzheimer’s disease
ANOVA	analysis of variance
CaMKII	Calmodulin-dependent protein kinase II
CCI	Controlled cortical impact
CDK1	Cyclin-dependent kinase 1
EA	Electroacupuncture
FPI	Fluid percussion injury
IPSC	Inhibitory postsynaptic currents
mNSS	modified neurological severity score
NORT	Novel object recognition test
PD	Parkinson’s disease
PSD	Postsynaptic density
qPCR	quantitative Real-Time PCR
ROCK	Rho-associated protein kinase
TBI	Traumatic brain injury
TEM	Transmission electron microscopy
WB	Western blotting

## References

[1] Han Q., Brenes D., Bishop K.W., Pichon T.J., Ling M., McPheron G.D., White N.J., Pun S.H., Liu J.T.C., Sellers D.L. (2025). Delivery of a fibrin-binding hemostatic polymer ameliorates neurovascular damage and neural tissue loss after traumatic brain injury. Sci. Adv..

[2] Huang J., Wang W., Peng Y., Weng W., Wei H., Feng Q., Wang A., Hu M., Li Z., Sun S. (2025). Neurophilic Biomimetic Lipoprotein-Mediated Targeted Nerve Growth Factor Delivery for Traumatic Brain Injury Therapy. Adv. Sci..

[3] Maas A.I.R., Menon D.K., Manley G.T., Abrams M., Åkerlund C., Andelic N., Aries M., Bashford T., Bell M.J., Bodien Y.G. (2022). Traumatic brain injury: Progress and challenges in prevention, clinical care, and research. Lancet Neurol..

[4] Li N., Lu W., Tang L., Zhu L., Deng W., Liu H., Huang C., Jin J., Zeng J., Chen S. (2025). Microglia in Post-Traumatic Brain Injury (TBI) Cognitive Impairment: From Pathological Changes to Therapeutic Approaches. CNS Neurosci. Ther..

[5] Lucke-Wold B.P., Logsdon A.F., Nguyen L., Eltanahay A., Turner R.C., Bonasso P., Knotts C., Moeck A., Maroon J.C., Bailes J.E. (2018). Supplements, nutrition, and alternative therapies for the treatment of traumatic brain injury. Nutr. Neurosci..

[6] Arizanovska D., Bush G.F., Dallera C.A., Folorunso O.O., Frye J.B., Doyle K.P., Jagid J.R., Wolosker H., Monaco B.A., Graciolli Cordeiro J. (2026). Cognitive loss after brain trauma results from sex-specific activation of synaptic pruning processes. Brain.

[7] Jamjoom A.A.B., Rhodes J., Andrews P.J.D., Grant S.G.N. (2021). The synapse in traumatic brain injury. Brain.

[8] Liu Q., Tan F., Wen J., Ma C., Wang Z., Fan X. (2026). Electroacupuncture ameliorates mitochondrial dysfunction and alleviates traumatic brain injury via the PANX1/ATP/Ca2+ pathway. Behav. Brain Res..

[9] Scheff S.W., Price D.A., Hicks R.R., Baldwin S.A., Robinson S., Brackney C. (2005). Synaptogenesis in the hippocampal CA1 field following traumatic brain injury. J. Neurotrauma.

[10] Fronczak K.M., Li Y., Henchir J., Dixon C.E., Carlson S.W. (2021). Reductions in Synaptic Vesicle Glycoprotein 2 Isoforms in the Cortex and Hippocampus in a Rat Model of Traumatic Brain Injury. Mol. Neurobiol..

[11] Alawieh A., Chalhoub R.M., Mallah K., Langley E.F., York M., Broome H., Couch C., Adkins D., Tomlinson S. (2021). Complement Drives Synaptic Degeneration and Progressive Cognitive Decline in the Chronic Phase after Traumatic Brain Injury. J. Neurosci..

[12] Empl L., Chovsepian A., Chahin M., Kan W.Y.V., Fourneau J., Van Steenbergen V., Weidinger S., Marcantoni M., Ghanem A., Bradley P. (2022). Selective plasticity of callosal neurons in the adult contralesional cortex following murine traumatic brain injury. Nat. Commun..

[13] Basu J., Siegelbaum S.A. (2015). The Corticohippocampal Circuit, Synaptic Plasticity, and Memory. Cold Spring Harb. Perspect. Biol..

[14] Hoffman A.N., Watson S., Fanselow M.S., Hovda D.A., Giza C. (2021). Region-Dependent Modulation of Neural Plasticity in Limbic Structures Early after Traumatic Brain Injury. Neurotrauma Rep..

[15] Lu J., Di Florio D.N., Boya P., Maday S., Springer W., Chu C.T. (2025). Autophagy and mitophagy at the synapse and beyond: Implications for learning, memory and neurological disorders. Autophagy.

[16] Thapak P., Ying Z., Gomez-Pinilla F. (2025). Enduring Effects of Humanin on Mitochondrial Systems in TBI Pathology. Biomolecules.

[17] Gobbel G.T., Bonfield C., Carson-Walter E.B., Adelson P.D. (2007). Diffuse alterations in synaptic protein expression following focal traumatic brain injury in the immature rat. Childs Nerv. Syst..

[18] Gao H., Jensen K., Nesbitt J., Ostroot M., Cary G.A., Wiley J., Trushin S., Watzlawik J.O., Springer W., Galkin A. (2025). Mitochondrial complex I deficiency induces Alzheimer’s disease-like signatures that are reversible by targeted therapy. Alzheimer’s Dement. J. Alzheimer’s Assoc..

[19] Olatona O.A., Sterben S.P., Kansakar S.B.S., Symes A.J., Liaudanskaya V. (2025). Mitochondria: The hidden engines of traumatic brain injury-driven neurodegeneration. Front. Cell. Neurosci..

[20] Sridharan P.S., Koh Y., Miller E., Hu D., Chakraborty S., Tripathi S.J., Kee T.R., Chaubey K., Vázquez-Rosa E., Barker S. (2024). Acutely blocking excessive mitochondrial fission prevents chronic neurodegeneration after traumatic brain injury. Cell Rep. Med..

[21] Svirsky S.E., Henchir J., Li Y., Carlson S.W., Dixon C.E. (2024). Temporal-Specific Sex and Injury-Dependent Changes on Neurogranin-Associated Synaptic Signaling After Controlled Cortical Impact in Rats. Mol. Neurobiol..

[22] Kong L., Yang H., Yang J., Jiang L., Xu B., Yang T., Liu W. (2025). Role of calcium overload-mediated disruption of mitochondrial dynamics in offspring neurotoxicity due to methylmercury exposure during pregnancy and lactation. Ecotoxicol. Environ. Saf..

[23] Xu S., Wang P., Zhang H., Gong G., Gutierrez Cortes N., Zhu W., Yoon Y., Tian R., Wang W. (2016). CaMKII induces permeability transition through Drp1 phosphorylation during chronic β-AR stimulation. Nat. Commun..

[24] Lim J.R., Lee H.J., Jung Y.H., Kim J.S., Chae C.W., Kim S.Y., Han H.J. (2020). Ethanol-activated CaMKII signaling induces neuronal apoptosis through Drp1-mediated excessive mitochondrial fission and JNK1-dependent NLRP3 inflammasome activation. Cell Commun. Signal. CCS.

[25] Hung S.Y., Chung H.Y., Luo S.T., Chu Y.T., Chen Y.H., MacDonald I.J., Chien S.Y., Kotha P., Yang L.Y., Hwang L.L. (2022). Electroacupuncture improves TBI dysfunction by targeting HDAC overexpression and BDNF-associated Akt/GSK-3β signaling. Front. Cell Neurosci..

[26] Cao L.X., Lin S.J., Zhao S.S., Wang S.Q., Zeng H., Chen W.A., Lin Z.W., Chen J.X., Zhu M.M., Zhang Y.M. (2023). Effects of acupuncture on microglial polarization and the TLR4/TRIF/MyD88 pathway in a rat model of traumatic brain injury. Acupunct. Med. J. Br. Med. Acupunct..

[27] Zhao S., Wang S., Cao L., Zeng H., Lin S., Lin Z., Chen M., Zhu M., Pang Z., Zhang Y. (2023). Acupuncture promotes nerve repair through the benign regulation of mTOR-mediated neuronal autophagy in traumatic brain injury rats. CNS Neurosci. Ther..

[28] Xiao L.Y., Wang X.R., Yang Y., Yang J.W., Cao Y., Ma S.M., Li T.R., Liu C.Z. (2018). Applications of Acupuncture Therapy in Modulating Plasticity of Central Nervous System. Neuromodulation.

[29] Sun Z., Zhang X., Li M., Yang Q., Xiao X., Chen X., Liang W. (2024). Targeting ferroptosis in treating traumatic brain injury: Harnessing the power of traditional Chinese medicine. Biomed. Pharmacother..

[30] Wang Z.N., Ding J.R., Li X., Shi L., Yin B., Bai G.H., Fang M., Lao L.X., Tian J., Bai L.J. (2025). Acupuncture Improves MRI Brain Microstructure with Postconcussion Symptoms in Mild TBI: A Randomized Controlled Trial. Radiology.

[31] Wei P., Wang K., Luo C., Huang Y., Misilimu D., Wen H., Jin P., Li C., Gong Y., Gao Y. (2021). Cordycepin confers long-term neuroprotection via inhibiting neutrophil infiltration and neuroinflammation after traumatic brain injury. J. Neuroinflamm..

[32] Yang D., Wu X., Yao Y., Duan M., Wang X., Li G., Guo A., Wu M., Liu Y., Zheng J. (2025). An RNA editing strategy rescues gene duplication in a mouse model of MECP2 duplication syndrome and nonhuman primates. Nat. Neurosci..

[33] Ho M.H., Tsai Y.J., Chen C.Y., Yang A., Burnouf T., Wang Y., Chiang Y.H., Hoffer B.J., Chou S.Y. (2024). CCL5 is essential for axonogenesis and neuronal restoration after brain injury. J. Biomed. Sci..

[34] Chen X., Chen C., Fan S., Wu S., Yang F., Fang Z., Fu H., Li Y. (2018). Omega-3 polyunsaturated fatty acid attenuates the inflammatory response by modulating microglia polarization through SIRT1-mediated deacetylation of the HMGB1/NF-κB pathway following experimental traumatic brain injury. J. Neuroinflamm..

[35] Xue Y., Zhang Y., Wu Y., Zhao T. (2024). Activation of GPER-1 Attenuates Traumatic Brain Injury-Induced Neurological Impairments in Mice. Mol. Neurobiol..

[36] Wan C., Xia Y., Yan J., Lin W., Zheng Y., Lin Y., Yao L., Chen Y. (2026). Restoration of excitation-inhibition balance and improvement of schizophrenia-like behavioral deficits via electroacupuncture. CNS Neurosci. Ther..

[37] Guo R.B., Dong Y.F., Yin Z., Cai Z.Y., Yang J., Ji J., Sun Y.Q., Huang X.X., Xue T.F., Cheng H. (2022). Iptakalim improves cerebral microcirculation in mice after ischemic stroke by inhibiting pericyte contraction. Acta Pharmacol. Sin..

[38] Luo B.L., Zhang Z.Z., Chen J., Liu X., Zhang Y.M., Yang Q.G., Chen G.H. (2023). Effects of gestational inflammation on age-related cognitive decline and hippocampal Gdnf-GFRα1 levels in F1 and F2 generations of CD-1 Mice. BMC Neurosci..

[39] Elser H., Magee R.G., Abbruzzese S., Sandsmark D., Gottesman R.F., Mosley T.H., Wolk D.A., Schneider A.L.C. (2025). Traumatic Brain Injury and Depressive Symptoms in Community-Dwelling Older Adults: A Prospective Cohort Study. Neurology.

[40] Rehman R., Froehlich A., Olde Heuvel F., Elsayed L., Boeckers T., Huber-Lang M., Morganti-Kossmann C., Roselli F. (2024). The FGFR inhibitor Rogaratinib reduces microglia reactivity and synaptic loss in TBI. Front. Immunol..

[41] Lin K., Chen J., Pan J., Wang R., Wu S., Wen W., Li Y., Wang L., Yuan F. (2025). Electro-Acupuncture to Treat Disorder of Consciousness (AcuDoc): Study Protocol for a Randomized Sham-Controlled Trial. Brain Behav..

[42] Wu M., Song W., Teng L., Li J., Liu J., Ma H., Zhang G., Zhang J., Chen Q. (2024). Exploring the biological basis of acupuncture treatment for traumatic brain injury: A review of evidence from animal models. Front. Cell. Neurosci..

[43] Shi Z., Mao L., Chen S., Du Z., Xiang J., Shi M., Wang Y., Wang Y., Chen X., Xu Z.X. (2025). Reversing Persistent PTEN Activation after Traumatic Brain Injury Fuels Long-Term Axonal Regeneration via Akt/mTORC1 Signaling Cascade. Adv. Sci..

[44] Kundu S., Singh S. (2023). What Happens in TBI? A Wide Talk on Animal Models and Future Perspective. Curr. Neuropharmacol..

[45] Siebold L., Obenaus A., Goyal R. (2018). Criteria to define mild, moderate, and severe traumatic brain injury in the mouse controlled cortical impact model. Exp. Neurol..

[46] Nuthalapati P., Holmes S.E., Altalib H.H., Fesharaki-Zadeh A. (2025). Synaptic Pathology in Traumatic Brain Injury and Therapeutic Insights. Int. J. Mol. Sci..

[47] Carson R.E., Naganawa M., Toyonaga T., Koohsari S., Yang Y., Chen M.K., Matuskey D., Finnema S.J. (2022). Imaging of Synaptic Density in Neurodegenerative Disorders. J. Nucl. Med. Off. Publ. Soc. Nucl. Med..

[48] Nakamura T., Sharma A., Lipton S.A. (2025). Aberrant S-nitrosylation in the TCA cycle contributes to mitochondrial dysfunction, energy compromise, and synapse loss in neurodegenerative diseases. Neurother. J. Am. Soc. Exp. Neurother..

[49] Cheng X.T., Huang N., Sheng Z.H. (2022). Programming axonal mitochondrial maintenance and bioenergetics in neurodegeneration and regeneration. Neuron.

[50] Klemmensen M.M., Borrowman S.H., Pearce C., Pyles B., Chandra B. (2024). Mitochondrial dysfunction in neurodegenerative disorders. Neurother. J. Am. Soc. Exp. Neurother..

[51] Fries G.R., Saldana V.A., Finnstein J., Rein T. (2023). Molecular pathways of major depressive disorder converge on the synapse. Mol. Psychiatry.

[52] Blagov A.V., Sukhorukov V.N., Orekhov A.N., Sazonova M.A., Melnichenko A.A. (2022). Significance of Mitochondrial Dysfunction in the Progression of Multiple Sclerosis. Int. J. Mol. Sci..

[53] Chan D.C. (2020). Mitochondrial Dynamics and Its Involvement in Disease. Annu. Rev. Pathol..

[54] Jin J.Y., Wei X.X., Zhi X.L., Wang X.H., Meng D. (2021). Drp1-dependent mitochondrial fission in cardiovascular disease. Acta Pharmacol. Sin..

[55] Al Ojaimi M., Salah A., El-Hattab A.W. (2022). Mitochondrial Fission and Fusion: Molecular Mechanisms, Biological Functions, and Related Disorders. Membranes.

[56] Pandya J.D., Leung L.Y., Hwang H.M., Yang X., Deng-Bryant Y., Shear D.A. (2021). Time-Course Evaluation of Brain Regional Mitochondrial Bioenergetics in a Pre-Clinical Model of Severe Penetrating Traumatic Brain Injury. J. Neurotrauma.

[57] Zhang S., Wang Z., Mao J., Zhang A., Zhang S., Chen J. (2025). Metabolomics of cerebrospinal fluid following traumatic brain injury: Exploration of biomarkers for secondary injuries and severity. Neurotherapeutics.

[58] Hill R.L., Kulbe J.R., Singh I.N., Wang J.A., Hall E.D. (2018). Synaptic Mitochondria are More Susceptible to Traumatic Brain Injury-induced Oxidative Damage and Respiratory Dysfunction than Non-synaptic Mitochondria. Neuroscience.

[59] Tao Y., Zang J., Wang T., Song P., Zhou Z., Li H., Wang Y., Liu Y., Jie H., Kuang M. (2025). Obesity-associated macrophages dictate adipose stem cell ferroptosis and visceral fat dysfunction by propagating mitochondrial fragmentation. Nat. Commun..

[60] Chen Y., Gong K., Xu Q., Meng J., Long T., Chang C., Wang Z., Liu W. (2021). Phosphoglycerate Mutase 5 Knockdown Alleviates Neuronal Injury After Traumatic Brain Injury Through Drp1-Mediated Mitochondrial Dysfunction. Antioxid. Redox Signal.

[61] Wu Q., Gao C., Wang H., Zhang X., Li Q., Gu Z., Shi X., Cui Y., Wang T., Chen X. (2018). Mdivi-1 alleviates blood-brain barrier disruption and cell death in experimental traumatic brain injury by mitigating autophagy dysfunction and mitophagy activation. Int. J. Biochem. Cell Biol..

[62] Bayer K.U., Giese K.P. (2025). A revised view of the role of CaMKII in learning and memory. Nat. Neurosci..

[63] Jin B., Gao Y., Fu Y., Zhang S., Zhang K., Su Y. (2024). Electroacupuncture improves cognitive function in a rat model of mild traumatic brain injury by regulating the SIRT-1/PGC-1α/mitochondrial pathway. Chin. Med. J..

[64] Zhang X., Zhang B., Qin X., Tang L., Wang C. (2025). Electroacupuncture Ameliorates Postoperative Cognitive Dysfunction and Oxidative Stress via the SIRT1/FOXO1 Autophagy Pathway: An Animal Study. J. Cell Mol. Med..

[65] Mollayeva T., Mollayeva S., Colantonio A. (2018). Traumatic brain injury: Sex, gender and intersecting vulnerabilities. Nat. Rev. Neurol..

[66] Bah M.G., Naik A., Barrie U., Dharnipragada R., Eden S.V., Arnold P.M. (2023). Racial and gender disparities in traumatic brain injury clinical trial enrollment. Neurosurg. Focus..

