# Supplementary material for: Electroacupuncture Prevents TBI-Induced Synaptic Loss by Inhibiting CaMKII/Drp1-Dependent Mitochondrial Fission
Source: Biomolecules. 2026 Aug 11;16(8):1169. doi: 10.3390/biom16081169 (PMC13511455; doi:10.3390/biom16081169)

# Supplementary Figure S1

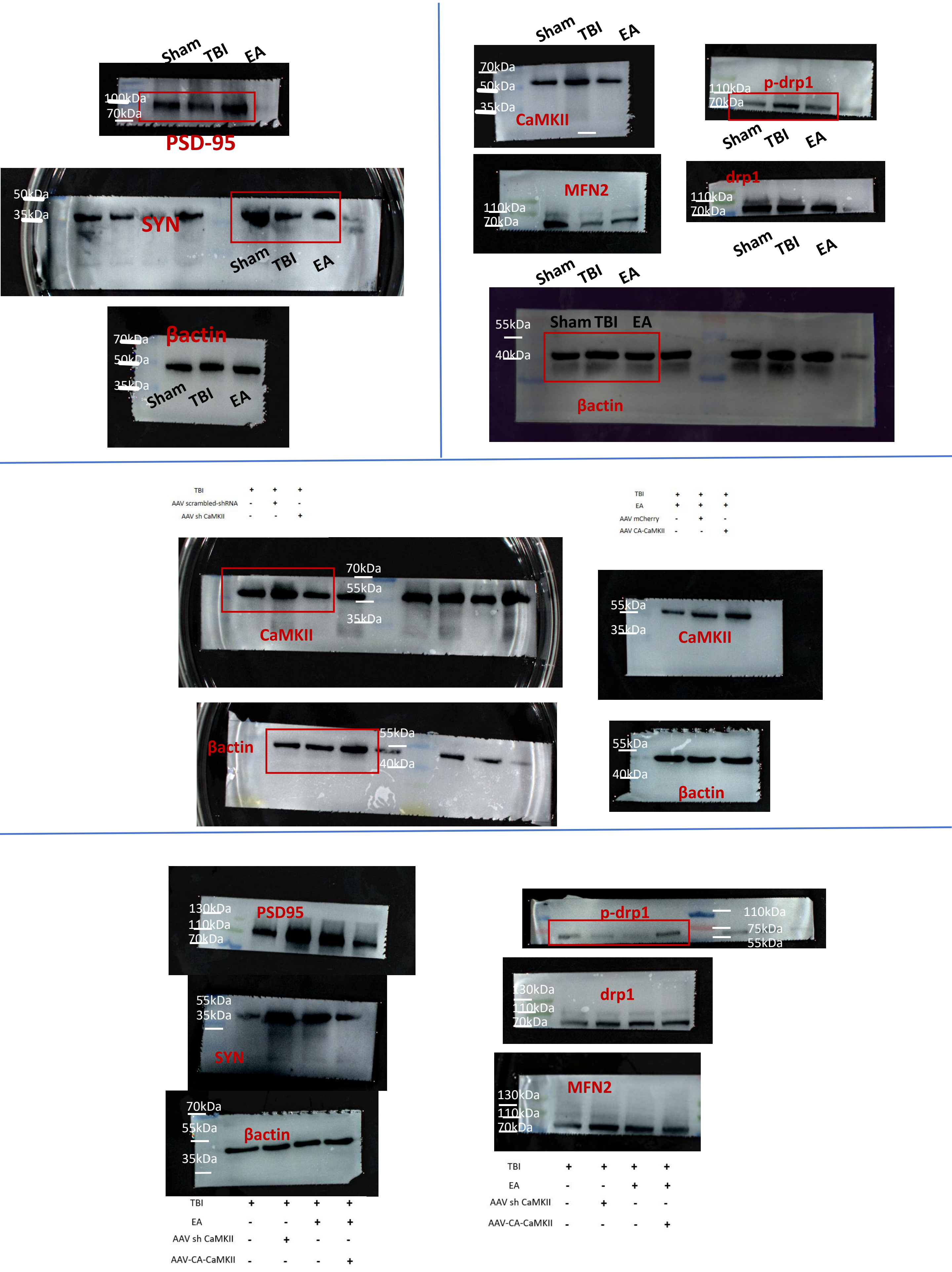

# Supplementary Figure S2

CamkII-βactin-Amplification

(A)

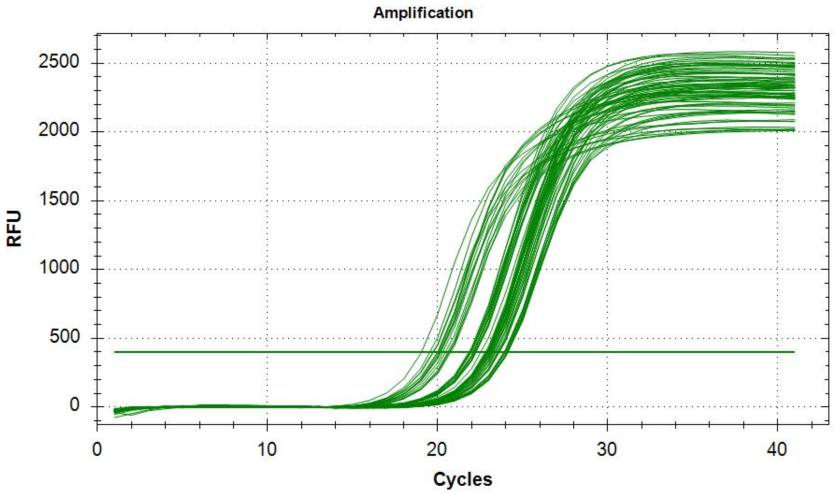


CamkII-βactin-Melt Curve

(B)

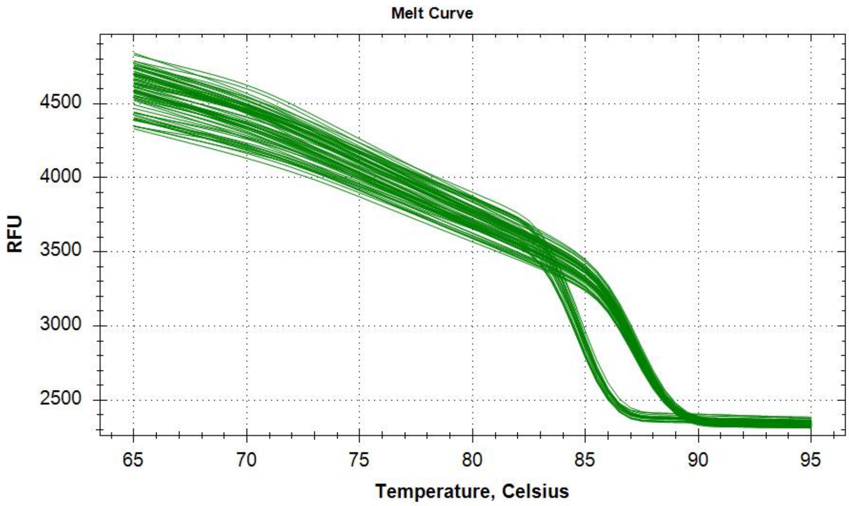


CamkII-βactin-Melt Peak

(C)

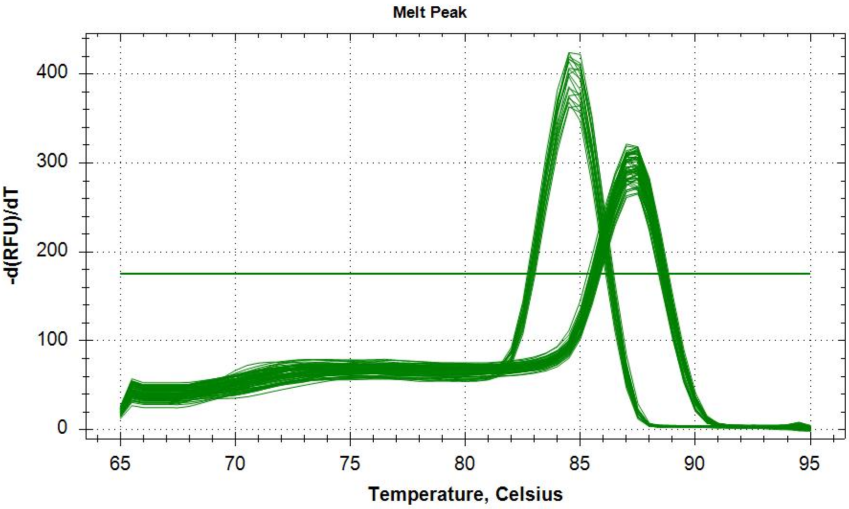

Supplement: Supplementary file 1 [file biomolecules-16-01169-s001.zip › biomolecules-4340791-supplementary.pdf]
